# El Niño and La Niña differentially drive transmission dynamics of the small ruminant parasite *Haemonchus contortus* across agroecological zones in Southern Africa

**DOI:** 10.1038/s41598-025-03156-8

**Published:** 2025-07-01

**Authors:** Jonathan Henry Ian Tinsley, Eric Rene Morgan

**Affiliations:** School of Biological Sciences, Queen’s University Belfast, 19 Chlorine Gardens, Belfast, BT9 5DL UK

**Keywords:** Biogeography, Climate change

## Abstract

The El Niño La Niña Southern Oscillation (ENSO) exerts a significant influence on agroecological environments and plays a crucial role in influencing crop productivity, yet its impact on animal diseases has been largely overlooked, despite the evident impact of climate on disease transmission. This study aimed to evaluate the impact of ENSO on the transmission potential (*Q*_*0*_) of *Haemonchus contortus (H. contortus)* across agroecological zones (AEZs) in southern Africa across the region’s typical rainy season (November-April). Key results indicate that stronger El Niño events can significantly decrease *H. contortus* transmission potential in subtropical AEZs. Tropical AEZs exhibit lower sensitivity to El Niño generally, but stronger events can lead to significant transmission potential reductions in certain zones. Conversely, La Niña increases transmission potential in tropical AEZs by small-to-medium effect magnitudes, depending on AEZ. Importantly, the analysis found significant increases in *Q*_*0*_ across direct transitions from El Niño to La Niña, with statistically significant medium effects observed in all tropical AEZs, except for the cool-subhumid AEZ. In subtropical AEZs, notable increases in *Q*_*0*_ were found in the warm-humid and warm-subhumid zones, also according to medium effect sizes. The study highlights the necessity for disseminating proactive and adaptive animal management practices for small ruminants among smallholder farmers, both during ENSO events and continuing into the post-event transition period, particularly through capacity building, nutritional supplementation and targeted selective treatment. The findings underscore the importance of considering small ruminants in ENSO preparedness and response plans, allowing farmers to optimise the resilience-enhancing roles of small ruminants to mitigate the impacts of climate shocks and food insecurity.

**Supplementary Information:**

The online version contains supplementary material available at 10.1038/s41598-025-03156-8.

## Introduction

The El Niño-La Niña Southern Oscillation (ENSO) is a principal driver of global climate interannual variability and has direct implications for agricultural production in southern Africa^1,2^. ENSO has three stages: neutral, El Niño and La Niña. El Niño typically leads to enhanced warming and significantly below-average precipitation anomalies across southern Africa, while La Niña typically results in significantly enhanced daily precipitation over all of Africa in February and much less so during the other five months of the typical rainy season (November-April) in southern Africa. Generally, the two phases do not display equal and opposite spatiotemporal evolutions of daily precipitation anomalies over southern Africa^3,4^, leading to stochastic and unpredictable weather variation.

ENSO affects smallholder farmer livelihoods in southern Africa by causing climate-driven agricultural production shocks that negatively affect food and economic security in the region^5^. Many studies have investigated the impact of ENSO on crop production^6–9^, meaning impacts on crop systems are well-documented. Crop farmers commonly integrate livestock, and especially goats, into their farming systems to mitigate the impact of crop-based production shocks, enhancing resilience and food security^10–13^. Yet livestock are prone to ENSO-derived production shocks as well, and there have been calls by the animal health research community for research assessing the regional impacts of ENSO on animal health: “This understanding of the potential impacts can then be used to develop plans that are locally relevant and engage key agencies and community stakeholders in planning and preparedness, as well as design communication strategies for different audiences” (WOAH, n.d.). To date, efforts have been made to assess the impact of ENSO on Rift valley fever^14–16^ and East Coast fever^17^. Surprisingly, studies seeking to assess the impact of ENSO on other livestock diseases, including those highly pertinent to smallholder systems, appear to be lacking.

Small ruminants are livestock assets commonly owned by resource-poor farmers in southern Africa and frequently suffer morbidity and mortality as a result of endemic gastrointestinal nematode (GIN) parasitic diseases. This is especially the case with *Haemonchus contortus* (*H. contortus*), a haematophagous GIN which strongly constrains small ruminant production across Africa^18–20^, exacerbating food and economic insecurity.

In its free-living stages, the development and survival of *H. contortus* is strongly influenced by climatic conditions^21–28^. The *Q*_*0*_ model, as previously applied by^29–31^, is a deterministic, environmentally stochastic climate-parasite model that estimates changes in *H. contortus* transmission potential in relation to daily climatic conditions. The basic reproductive quotient, *Q*_*0*_, is analogous to the basic reproductive ratio, *R*_*0*_, which describes the predicted number of newly infected hosts resulting from a typical infected individual during its period of infectiousness, based on a population of susceptibles only^32–34^. *R*_*0*,_ however, is not applicable to the dynamics of *H. contortus* since it assumes infections to be autonomous processes where within-host multiplication occurs so quickly that additional reinfections have less significant implications^32^. This is not the case with *H. contortus* however, which instead characterises as an inherently nonautonomous infection, whereby infection load is entirely dependent on ingestion of additional infective larvae. *Q*_*0*_ forecasts the anticipated quantity of eggs produced by a solitary adult female during its lifespan that successfully hatch, progress to stage 3 infective larvae (L3), are ingested, and ultimately mature into adult worms. These predictions are driven by daily climatic conditions and, similarly to *R*_*0*_, assume the absence of density-dependent constraints^31,32^.

This study therefore seeks to investigate the impact of ENSO on *H. contortus* transmission potential in southern Africa across the region’s agroecological zones (AEZs). Outcomes intend to improve our understanding of *H. contortus* infection dynamics during ENSO in southern Africa, and to support the targeted integration of small ruminant health management strategies into preparation and response plans.

## Materials and methodology

### The *Haemonchus contortus Q*_*0*_ model

The *Q*_*0*_ model is driven by Eq. 1:

1 $$\:{Q}_{0\:}=\:\frac{q\lambda\:}{\mu\:}\frac{\beta\:p}{\rho\:+\beta\:H}H{m}_{2}\:\:\:$$

This equation, from Rose, et al.^29^, is based on the original equation for *Q*_*0*_ by Heesterbeek and Roberts^32^, with parameters given below and in Table 1. As noted by Rose, et al.^29^, the equation was subsequently extended by Bolajoko, et al.^30^ to incorporate climate dependence in the life-history of free-living stages and to add a horizontal larval migration parameter (Eq. 2):

2 $$\:q=\:\frac{\delta\:{m}_{1}}{({\mu\:}_{e}+\:\delta\:)({\mu\:}_{l3}+\:{m}_{1})}\:\:\:$$

Here, *q* represents the probability of an egg developing to L3 and successfully migrating onto herbage. Specifically, this is estimated based on non-linear interactions between the development rate of eggs to L3 (*δ*), the horizontal migration of L3 from faeces to herbage (*m*_*1*_*)*, the egg mortality rate (*µ*_e_) and larval survival rate in faeces (*µ*_L3_). The model will only produce a value for *q* if the nine-day cumulative precipitation average (P), divided by the nine-day cumulative potential evapotranspiration average (PET), is more than or equal to 1. When this condition is satisfied, *q* is estimated as a function of temperature, and when P/PET is less than one, the model will produce a *q* value of zero^29^.

Given *q* > 0, *Q*_*0*_, as described in Eq. 1 and by Rose, et al.^29^, refers to: (1) the number of eggs that successfully develop into L3 that are produced by a single adult female in her lifetime, based on: the fecundity rate (*λ)*, adult mortality (*µ)*, the probability of an egg developing to L3 and successfully migrating onto herbage (*q*, as described above), and (2) the number of adult worms that are successfully produced by each L3, which is estimated based on: the establishment rate of ingested L3 within host (*p);* the L3 mortality rate within the larval environment (*ρ*); host L3 ingestion rate (*β)*; host density (*H*); and *m*_*2*_, which is an equation extension by Rose, et al.^29^ to represent a vertical migration parameter, reflecting fluctuating L3 distributions between soil and herbage in the larval environment. For exact parameter definitions and estimates, see Table 1. By holding *p*,* β* and *H* constant, results from Eq. 1 indicate the effect of climatic conditions on transmission potential, independent of animal factors.

**Table 1:** *Q*_*0*_ model parameters, definitions, values and sources. Adapted from^29^.

Parameter	Definition	Values	Source
Haemonchus contortus			
λ	Fecundity (eggs day − 1 per adult)	2250	^35^
µ	Instantaneous daily mortality rate of adult nematodes	0.05	^36^
*q*	Probability that an egg will develop to L3 and migrate onto pasture	$\:q=\:\frac{\delta\:{m}_{1}}{({\mu\:}_{e}+\:\delta\:)({\mu\:}_{l3}+\:{m}_{1})}\:\:\:\:\:\:\:\:\:$	^37–39^
*δ*	Instantaneous daily development rate of eggs to L3	$\:-0.09746\:+\:0.01063Tmean$	^39^
*µ* _*e*_	Instantaneous daily mortality rate of eggs	$\:\text{exp}\left(-1.3484\:\--\:0.10488Tmean\:+\:0.00230Tmeanl2\right)$	^39^
*µ* _*l*3_	Instantaneous daily mortality rate of L3 in faeces	$\:\text{e}\text{x}\text{p}(-2.62088\:\--\:0.14399Tmean\:+\:0.00462Tmean2$	^39^
*m* _*1*_	Instantaneous daily L3 migration rate between faeces and pasture	0.25	^39^
*ρ*	Instantaneous daily mortality rate of L3 on pasture	$\:\frac{\mu\:l3}{3}$	^30^
*m* _*2*_	Proportion of total pasture L3 that are found on herbage	0.2	^22,40^
*p*	Probability of establishment of ingested L3	0.4	^41^
Host management			
*β*	Rate of ingestion of L3 on pasture	$\:\frac{c}{BA}$	-
*c*	Daily herbage dry matter intake per host (kg DM day^− 1^)	1.4	^42^
*H*	Host density or stocking density (per ha)	Held constant, 1	-
*B*	Standing biomass (kg DM ha^− 1^)	2000	^42,43^
*A*	Grazing area (ha)	Held constant, 1	-
Climate			
*d*	Dekad	-	Standard dekad
*P*	Total daily precipitation (mm)	Daily variable	^44^
*Pt*	Saturated water vapour density at the daily mean temperature (g/m^3^)	$\:\frac{Pt\:=\:4.95\text{exp}\left(0.062\:T\:mean\right)}{100}$	^45^
*PET*	Daily potential evapotranspiration (mm day^− 1^)	$\:0.55\:\left({\left(\frac{photoperiod}{12}\right)}^{2}\right)Pt\:25.4$	^45^
*T* _mean_	Mean daily temperature (°C)	Daily variable $\:\frac{T\:\text{max}+T\:min}{2}$	-
*T* _min_	Minimum daily temperature (°C)	Daily variable	^46^
*T* _*max*_	Maximum daily temperature (°C)	Daily variable	^46^

### Climate datasets

The *Q*_*0*_ model generates values based on climatic conditions, utilising total daily precipitation and minimum and maximum temperature. The daily temperature data employed within this study was sourced from the Climate Hazards Center Infrared Temperature with Stations (CHIRTS-daily) 0.05 degrees resolution dataset^46,47^. The daily precipitation data used was obtained from the Climate Hazards Center Infrared Precipitation with Stations (CHIRPS) Africa-daily 0.05 degrees resolution dataset^44,48^. All climate data was pre-processed to only include data for southern Africa and across the analysis period 1987–2016. Pre- and post-processing of geospatial data was undertaken using Climate Data Operators (CDO)^49^. The rainy season defines the transmission season for *H. contortus* in southern Africa and typically spans from November to April, hence data was only analysed over this period. This was necessary as the fine resolution datasets employed to run the model led to computational constraints, which limited the boundaries of the analysis. A comprehensive analysis of the climate data employed in this study is presented by Hoell, Gaughan, Magadzire and Harrison^3^.

### Analytical approach

**Fig. 1:**
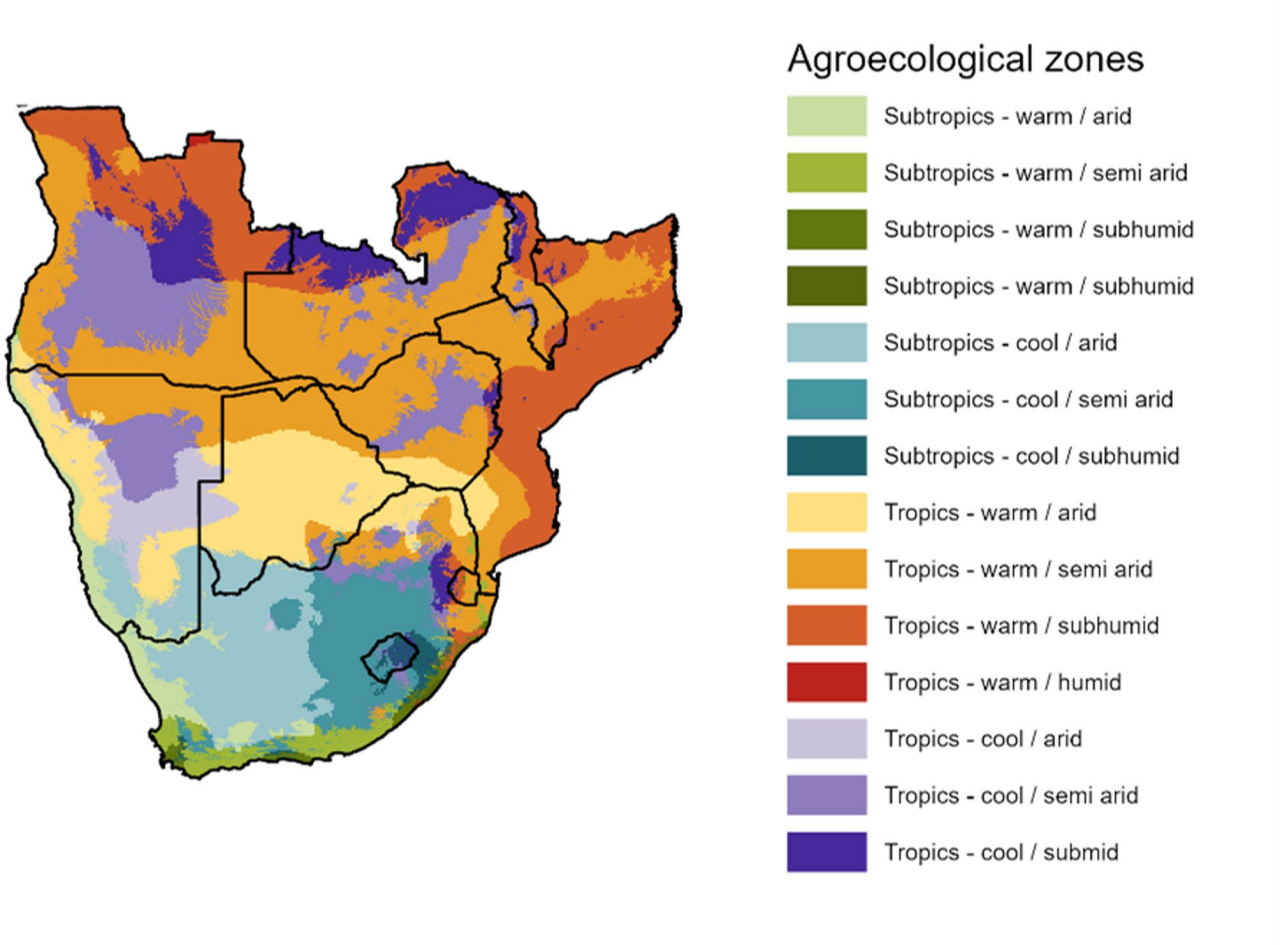
Agroecological zones (AEZs) of southern Africa used to segment the analysis. Source: Author using ArcGIS Pro, data from^51^.

In order to segment the spatiotemporal *Q*_*0*_ data for time series analysis, data was extracted by AEZ in ArcGIS Pro using the ‘Zonal Statistics’ Spatial Analyst tool^50^. Figure 1 shows the AEZs in southern Africa used to extract mean *Q*_*0*_ values. AEZ data was sourced from Sebastian^51^.

Although AEZs were originally created for agronomic purposes, the factors governing crop growth in Africa are similar to those governing *H. contortus* transmission dynamics. Warm and moist environmental conditions are critical the development rate of eggs to L3^21^ and their subsequent migration onto herbage (Wang et al., 2014). Subtropical AEZs are classified as such if the mean monthly temperature (adjusted to sea level) is less than 18 degrees Celsius for one or more months, whereas for tropical AEZs the mean monthly temperature must be greater than 18 degrees Celsius for all months of the year^51^. This is also in line with *Haemonchus* development dynamics, where temperatures of lower than 18 degrees Celsius are associated with increasingly lower success rates of egg development to L3^52^.

Moisture zones used to categorise AEZs are derived from the length of the growing period (LGP), which is calculated based on the period of time when both moisture and temperature are conducive to crop growth^51^. The LGP is estimated as the annual period where temperatures are greater than or equal to 5 degrees Celsius and precipitation plus moisture stored in the soil are greater than half the potential evapotranspiration. A normal growing period is where precipitation exceeds the potential evapotranspiration rate, thus providing vegetation with the moisture required for evapotranspiration while replenishing the soil moisture profile^51^. This compliments the *Q*_*0*_ model given the model calculates *q* based on whether precipitation exceeds PET^29^. Such conditions provide the moisture required for larval development within faeces and subsequent migration first out of faeces, and then between the soil and vegetation column^23–26,28,53^.

Arid AEZs are defined as having less than 70 days LGP, semiarid 70–180 days LGP, subhumid 180–270 LGP, and humid > 270 LPG^51^. AEZs are further classified as warm or cool, referring to highland or lowland areas in reflection of the impact of elevation on temperatures. For the tropics, areas with > 1200 m elevation are classified as cool, whereas areas with > 800 m elevation define cool areas for subtropical AEZs^51^. This aspect of the AEZ methodology is also congruent with the *Q*_*0*_ model given the impact of elevation on temperature, and of temperature on development, as shown within Rose, et al.^29^.

Once *Q*_*0*_ values for each AEZ were extracted, data from Hoell, Gaughan, Magadzire and Harrison^3^ was used to categorise southern Africa rainy season months (November to April) between 1987 and 2016 as neutral, El Niño, or La Niña (see Table 2). A transition is defined by the months in which an ENSO phase shifts from one to the other (e.g. from El Niño to La Niña, La Niña to neutral, or El Niño to neutral). Subsequent analysis presented in the results section was conducted and visualised in R Studio Posit^54^, while geospatial anomaly analysis was conducted using ESRI^50^ Image Analyst tools.

**Table 2:** List of El Niño and La Niña events between 1987 and 2016 on Southern Africa.

Month	Neutral	El Niño	La Niña
November	1989, 1990, 1992, 1993, 1996, 2001, 2003, 2012, 2013	1987, 1991, 1994, 1997, 2002, 2004, 2006, 2009, 2014, 2015	1988, 1995, 1998, 1999, 2000, 2005, 2007, 2008, 2010, 2011, 2016
December	1988, 1989, 1990, 1992, 1993, 1996, 2001, 2003, 2012, 2013	1987, 1991, 1994, 1997, 2002, 2004, 2006, 2009, 2014, 2015	1995, 1998, 1999, 2000, 2005, 2007, 2008, 2010, 2011, 2016
January	1990, 1991, 1993, 1994 1997, 2002, 2004, 2013, 2014	1987, 1988, 1992, 1995, 1998, 2003, 2005, 2007, 2010, 2015, 2016	1989, 1996, 1999, 2000, 2001, 2006, 2008, 2009, 2011, 2012
February	1990, 1991, 1993, 1994, 1997, 2002, 2004, 2007, 2013, 2014	1987, 1988, 1992, 1995, 1998, 2003, 2005, 2010, 2015, 2016	1989, 1996, 1999, 2000, 2001, 2006, 2008, 2009, 2011, 2012
March	1988, 1990, 1991, 1993, 1994, 1997, 2001, 2002, 2003, 2004, 2005, 2007, 2013, 2014	1987, 1992, 1995, 1998, 2010, 2015, 2016	1989, 1996, 1999, 2000, 2006, 2008, 2009, 2011, 2012
April	1988, 1990, 1991, 1993 1994, 1995, 1996, 1997 2001, 2002, 2003, 2004 2005, 2006, 2007, 2009 2010, 2012, 2013, 2014	1987, 1992, 1998, 2015, 2016	1989, 1999, 2000, 2008, 2011

Table source: Author adaptation of Hoell, Gaughan, Magadzire and Harrison^3^.

To assess the impact of El Niño and La Niña on *H. contortus* transmission potential, Welch’s t-tests were used to analyse the relationships across AEZs between rainy season *Q*_*0*_ values across the three different phase pairings: Neutral-El Niño, Neutral-La Niña, and El Niño-La Niña. Statistical testing showed that the *Q*_*0*_ data displayed a mix of characteristics across AEZs and ENSO phase pairings, with both normal and non-normal distributions, as well as equal and unequal variances depending on the comparison. Sample sizes were consistently unequal across phases. Welch’s t-test is particularly useful for comparing the means of two groups with unequal sample sizes and when the equal variance assumption is violated. Given the moderate to large sample sizes across groups, the Central Limit Theorem supports its validity under even non-normal distributions, ensuring greater statistical power compared to non-parametric alternatives which can be sensitive to unequal sample sizes and variances.  In order to distinguish the magnitude of changes assessed through the t-tests, percentage changes were calculated in addition to Cohen’s *d*. Cohen’s *d* permits an understanding of the practical significance of results and provides a standardised approach to enable an interpretation of the magnitude of differences between groups, remaining useful with small sample sizes^55^.

## Results

### Combined effects of ENSO on *Q*_*0*_ across agroecological zones

**Fig. 2:**
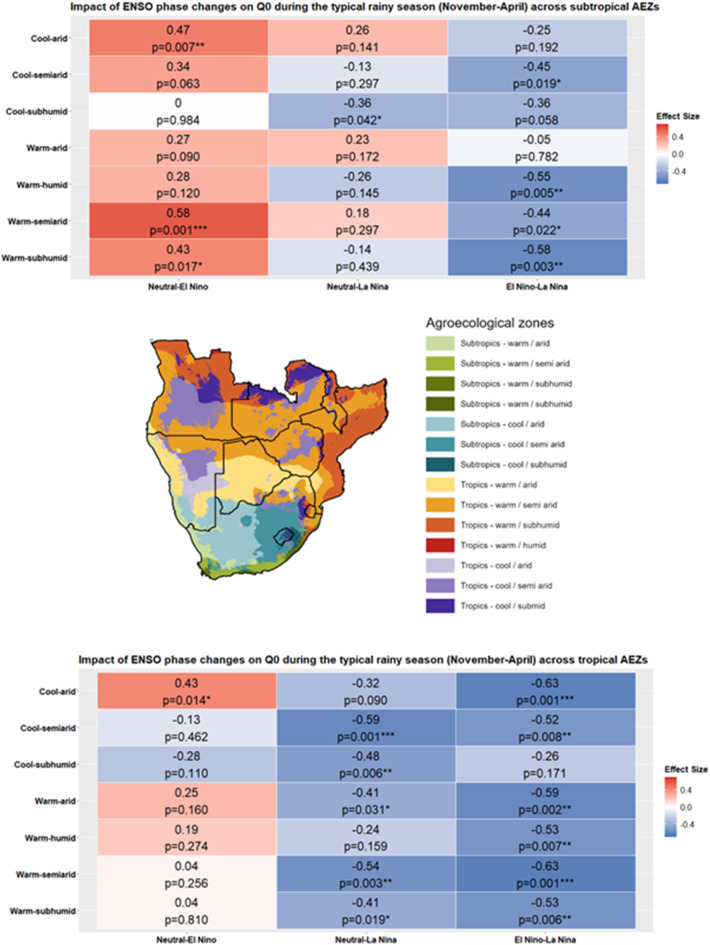
Effect sizes and statistical significance of ENSO phase transitions on Q0 during the typical southern Africa rainy season (November-April) across tropical and subtropical agroecological zones (AEZs). Red and blue tiles indicate positive and negative effect sizes respectively. Asterisks denote significance levels: * = *p* ≤ 0.05, ** = *p* ≤ 0.01, *** = *p* ≤ 0.001. Cohen’s *d* effect size classifications: negligible: *d* < 0.2, small: 0.2 ≤ *d* < 0.5, medium: 0.5 ≤ *d* < 0.8, large: *d* ≥ 0.8.

The relationship between ENSO phases and *H. contortus* transmission potential is shown in Fig. 2, with more detailed statistics presented in Table S1 in the Supplementary Materials. These are expressed in terms of differences in *Q*_*0*_ model outputs when moving from one ENSO phase to another, i.e. transitions between neutral, El Niño and La Niña. Classifications of effect size employ Cohen’s *d* classifications (negligible: *d* < 0.2, small: 0.2 ≤ *d* < 0.5, medium: 0.5 ≤ *d* < 0.8, large: *d* ≥ 0.8).

The heatmaps presented in Fig. 2 summarise mean *Q*_*0*_ differences across ENSO phases over the typical rainy season in southern Africa per AEZ. The El Niño–La Niña pairing yielded a notably high number of statistically significant results across 10 of the 14 AEZs, with eight of these ten exhibiting medium negative effect sizes (*d* = − 0.52 to − 0.65), indicating significantly lower mean *Q*_*0*_ during El Niño compared to La Niña. Mean *Q*_*0*_ was also significantly lower across six of the seven tropical AEZs for the same phase pairing, namely the cool-arid (*d* = -0.63, p-value = 0.001), cool-semi-arid (*d* = -0.52, p-value = 0.008), warm-arid (*d* = -0.59, p-value = 0.002), warm-semiarid (*d* = -0.63, p-value = 0.001), warm-humid (*d* = -0.53, p-value = 0.007), and warm-subhumid (*d* = -0.53, p-value = 0.006) AEZs, all of which were associated with medium negative effect sizes. Four subtropical AEZs yielded statistically significant results, although this was only associated with a medium negative effect size for the warm-humid (*d* = -0.55, p-value = 0.005) and warm-subhumid (*d* = 0.58, p-value = 0.003) AEZs.

Heatmap data for the Neutral-La Niña pairing indicates that *Q*_*0*_ did not significantly differ across subtropical AEZs for this pairing, except for the cool-subhumid AEZ (p-value = 0.042), although the effect size was small (*d* = -0.36). La Niña was shown to have a greater effect on *Q*_*0*_ compared to neutral phase values in tropical AEZs however, with statistically significant differences associated with a medium effect size observed for the cool-semiarid (*d* = -0.59, p-value = 0.001) and warm-semiarid (*d* = -0.54, p-value = 0.003) AEZs. Statistically significant differences in *H. contortus* transmission potential associated with a small effect size were observed in the cool-subhumid (*d* = -0.48, p-value = 0.006), warm-arid (*d* = 0.41, p-value = 0.031) and warm-subhumid (*d* = 0.41, p-value = 0.019) AEZs.

Transitions between neutral phases and El Niño did not commonly result in meaningful changes to *H. contortus* transmission potential in tropical AEZs, except for the cool-arid AEZ, although the effect size was small (*d* = 0.043, p-value= 0.014). *H. contortus* transmission potential was comparatively more impacted in subtropical AEZs for the same pairing. A significant difference equivalent to a medium positive effect size was detected for the warm-semiarid AEZ (*d* = 0.58, p-value =0.001), and while the effect size in both cases was small, statistically significant differences in *Q*_*0*_ were also observed across the warm-subhumid (*d* = 0.43, p-value = 0.017) and cool-arid (*d* = 0.47, p-value = 0.007) AEZs. This indicates El Niño reduces Q_0_ in these AEZs compared to neutral conditions.

**Fig. 3:**
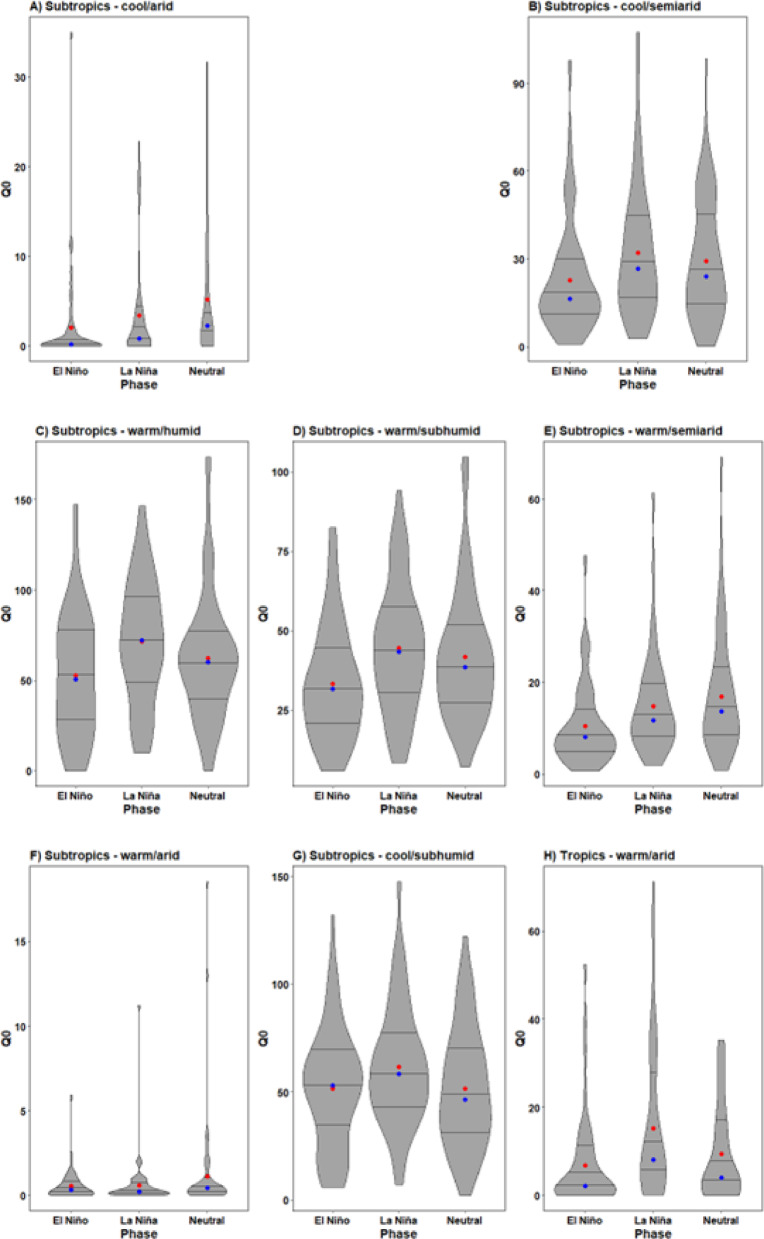

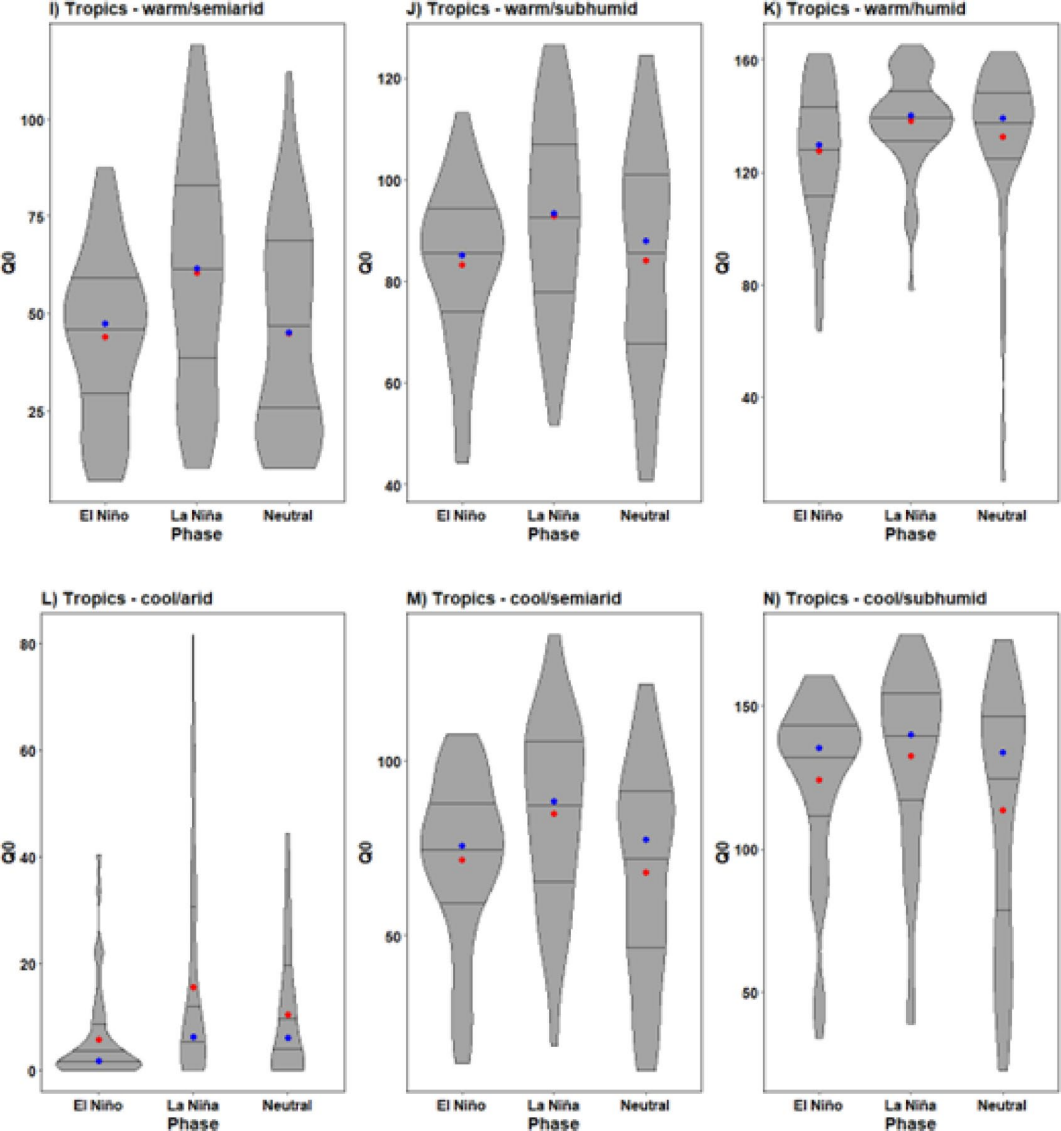
Violin plots of rainy season *Q*_*0*_ values across the seven different tropical and subtropical AEZs of southern Africa (A-N). Note the y-axis scale differs across plots due to the scale of differences in transmission potential. Red markers indicate the mean value, while blue indicates the median. Lines show the interquartile range.

What stands out in the violin plots within Fig. 3 (A-G) is the substantial variation in rainy season *Q*_*0*_ values across subtropical AEZs. Arid subtropical AEZs were characterised as displaying quantitatively smaller *Q*_*0*_ values and exhibit a smaller range of *Q*_*0*_ values across the transmission season compared to more humid AEZs. Furthermore, interpretation of the shape of violin plots in Fig. 3 (A-G) indicates a high probability of *Q*_*0*_ being distributed around zero in these AEZs compared to semi-arid, subhumid and humid subtropical AEZs during El Niño. Violin plots (H-N) show likewise to subtropical AEZs significant variations in transmission dynamics across tropical AEZs. *Q*_*0*_ values were lowest in more arid tropical AEZs and highest in warmer and more humid zones. During El Niño, in tropical arid and semiarid AEZs, there were an increasing number of occasions where *Q*_*0*_ = 0 across the rainy season compared to neutral, as indicated by the wider base, indicating a higher probability of zero values occurring. The mean exceeding the median *Q*_*0*_ values in plots H and L are the result of outliers inflating mean *Q*_*0*_ values. All other tropical AEZs in Fig. 3 (I-K, M-N) show transmission potential was always greatly in excess of *Q*_*0*_ *=* 1, reflective of conditions being highly conducive to development throughout the rainy season in these zones.

**Fig. 4:**
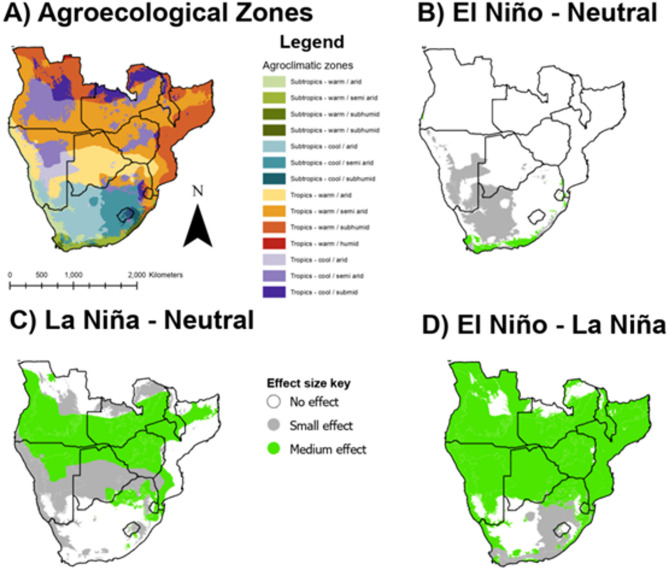
Area of effect map showing areas of southern Africa where a statistically significant difference in ENSO phase means was detected. For effect direction, refer to text and section “Specific ENSO events”.

The sum of these results suggests that the transition from El Niño to La Niña most substantially affects *H. contortus* transmission dynamics across each of the three ENSO phase pairings. The spatial distribution of all effect sizes is displayed in Fig. 4.

### Specific ENSO events

While the broad-scale effects presented in the above section provide a general indication of how ENSO impacts transmission dynamics in southern Africa AEZs, the inclusion of weak ENSO events in the pooled analysis likely moderates the impacts of more significant ENSO events. This subsection therefore presents analysis of the three strongest El Niño and La Niña events across 1987–2016 (as per NOAA^56^). For El Niño, the strongest events assessed were: (1) 2015/2016, (2) 1997/1998, and (3) 1991/1992. The strongest La Niña events include: (1) 2010/2012, (2) 2007/2009, and (3) 1998/2001. Note non-contiguous years reflect multi-year events. Welch’s t-test for unequal variance was used to assess significance of differences between means. Effect sizes were calculated using Cohen’s *d* with Hedges’ *g* correction to account for the small sample sizes, with monthly data points equating to less than 20 for some individual events.

#### Impacts of specific El Niño events

**Fig. 5:**
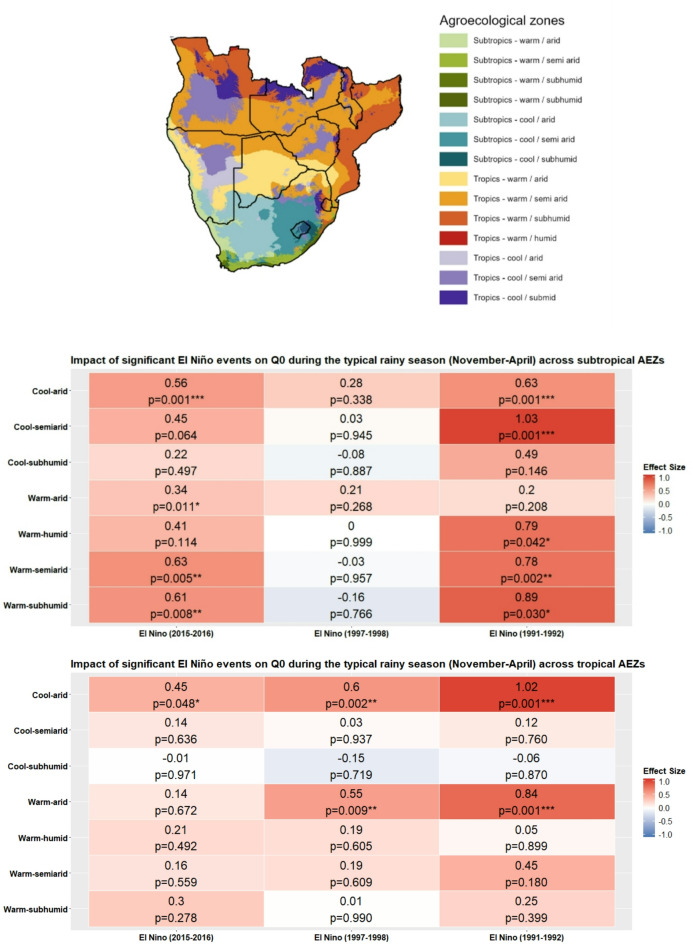
Effect sizes and statistical significance of differences of mean *Q*_*0*_ during major El Niño events compared to neutral phase values during the typical southern Africa rainy season (November-April) across tropical and subtropical agroecological zones (AEZs). Red and blue tiles indicate positive and negative effect sizes respectively. Asterisks denote significance levels: * = *p* ≤ 0.05, ** = *p* ≤ 0.01, *** = *p* ≤ 0.001. Cohen’s *d* effect size classifications: negligible: *d* < 0.2, small: 0.2 ≤ *d* < 0.5, medium: 0.5 ≤ *d* < 0.8, large: *d* ≥ 0.8. Note difference of legend scale compared to Fig. 2, reflective of higher detected effect sizes in this analysis.

Heatmap plots in Fig. 5 display effect sizes and p-values for differences in *Q*_*0*_ between strong El Niño events and neutral phases over the typical rainy season in southern Africa, stratified by AEZ. Table S2 in the Supplementary Materials presents detailed statistical analysis.

Overall, the 2015–2016 and 1991–1992 El Niño events were associated with widespread and statistically significant decreases of *Q*_*0*_ across AEZs. The 1991–1992 event was particularly impactful. Statistically significant decreases of *H. contortus* transmission potential equivalent to a large effect size were observed in the cool-arid (*d* = 1.03, p-value = 0.001) and warm-subhumid (*d* = 0.89, p = value = 0.030) subtropical AEZs, with large effects also associated with statistically significant decreases in the cool-arid (*d* = 1.02, p-value = 0.001) and warm-arid (*d* = 0.84, p-value = 0.001) tropical AEZs. Statistically significant decreases of *Q*_*0*_ were also observed in the warm-humid (*d* = 0.78, p-value = 0.030) and warm-semiarid (d = 0.78, p-value = 0.002) subtropical AEZs, both characterised by a medium effect size bordering on the conventional threshold for a large effect.

The 2015–2016 El Niño was also associated with *H. contortus* transmission potential perturbations of practical significance. Across subtropical AEZs, the cool-arid (*d* = 0.56, p-value = 0.001), warm-semiarid (*d* = 0.63, p-value = 0.005) and warm-subhumid (*d* = 0.61, p-value = 0.008) zones exhibited statistically significant decreases of *Q*_*0*_ equivalent to a medium effect size. Only the cool-arid tropical AEZ (*d* = 0.45, p-value = 0.048) yielded a statistically significant change in *Q*_*0*_, although of limited practical significance as emphasised by the small effect size.

Comparatively, the 1997–1998 event had minimal and non-significant impacts across AEZs, with exception to the cool-arid (*d* = 0.6, p-value = 0.002) and warm-arid (*d* = 0.55, p-value = 0.009) tropical AEZs.

#### Impacts of specific La Niña events

**Fig. 6:**
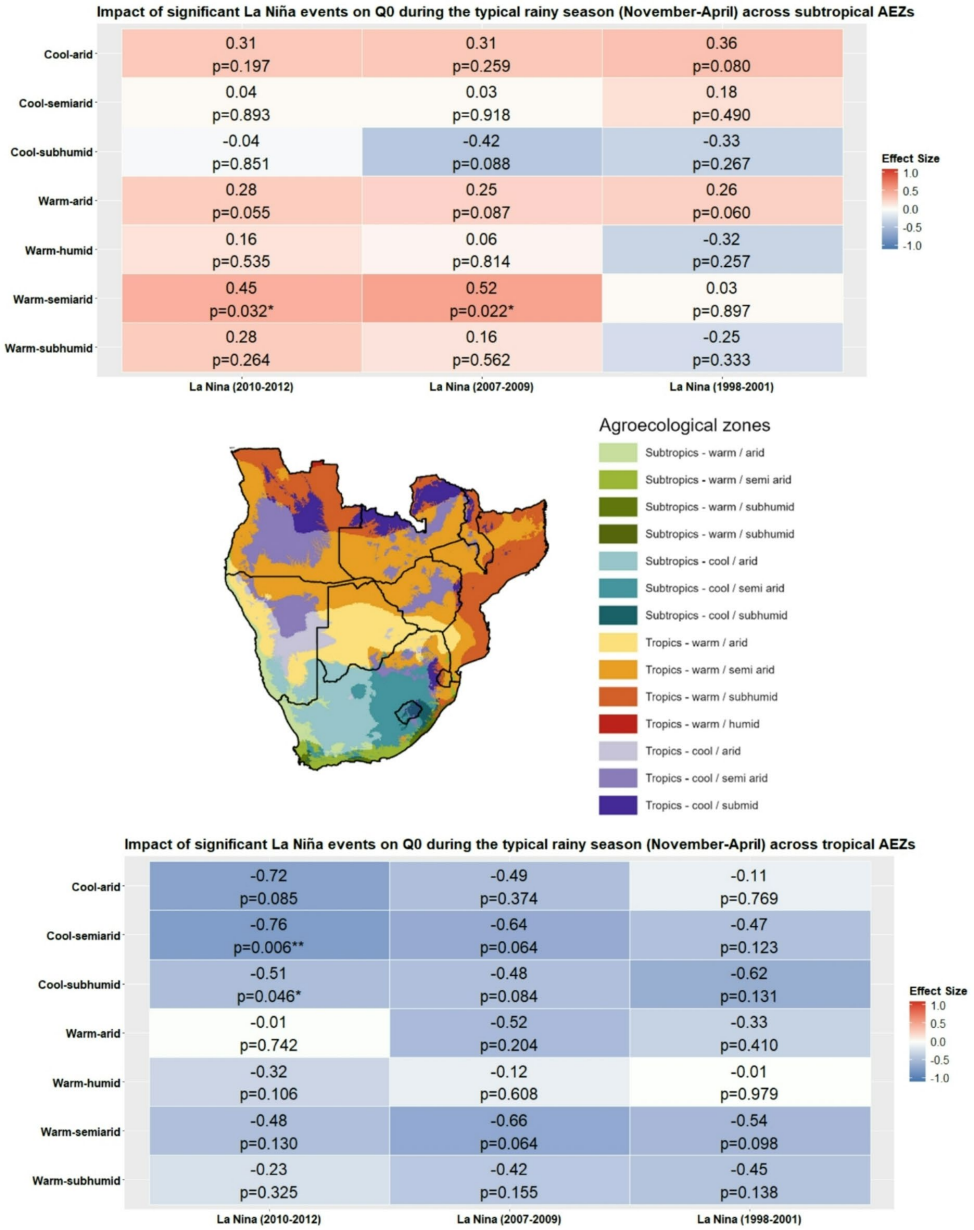
Effect sizes and statistical significance of differences in mean *Q*_*0*_ during major La Niña events compared to neutral phase values during the typical southern Africa rainy season (November-April) across tropical and subtropical agroecological zones (AEZs). Red and blue tiles indicate positive and negative effect sizes respectively. Asterisks denote significance levels: * = *p* ≤ 0.05, ** = *p* ≤ 0.01, *** = *p* ≤ 0.001. Cohen’s *d* effect size classifications: negligible: *d* < 0.2, small: 0.2 ≤ *d* < 0.5, medium: 0.5 ≤ *d* < 0.8, large: *d* ≥ 0.8. Note difference of legend scale compared to Fig. 2, reflective of higher detected effect sizes in this analysis.

The direction of effect across the analysed La Niña events was more variable compared to those of El Niño, as emphasised by the heatmap plot in Fig. 6 and data in Table S3 in the Supplementary Materials. La Niña led to few statistically significant changes in *Q*_*0*_ across all sampled events for subtropical AEZs, except for the warm-semiarid AEZ, which experienced a statistically significant decrease in the 2010–2012 event (*d* = 0.45, p-value = 0.032), equivalent to a small effect size, with the same zone experiencing a decrease equivalent to a medium effect in the 2007–2009 event (*d* = 0.52, p-value = 0.022).

The impact of La Niña on tropical AEZs was overwhelmingly reflective of an increase in *H. contortus* transmission potential, with Fig. 6 displaying the effect sizes and statistical significance of differences in *Q*_*0*_ between neutral conditions and the three major La Niña events. Overall, La Niña was associated with moderate increases in *Q*_*0*_ across multiple AEZs, although few of these were statistically significant, possibly a result of small sample sizes of monthly data points. Statistically significant increases in *Q*_*0*_ were observed in the cool-semiarid AEZ during the 2010–2012 La Niña (*d* = -0.76, *p* = 0.006), associated with a medium effect size that bordered on the threshold for a large effect size. Similarly, a significant medium effect was observed in the cool-subhumid AEZ during the same period (d = -0.51, *p* = 0.046). Across other AEZs, effect sizes were generally likewise reflective of higher *Q*_*0*_ during La Niña, although not statistically significant, and most fell within the small to medium effect size range. These findings suggest that La Niña conditions may lead to notable increases in *H. contortus* transmission potential in tropical AEZs.

#### Mapping *Q*_*0*_ anomalies across major ENSO events

**Fig. 7:**
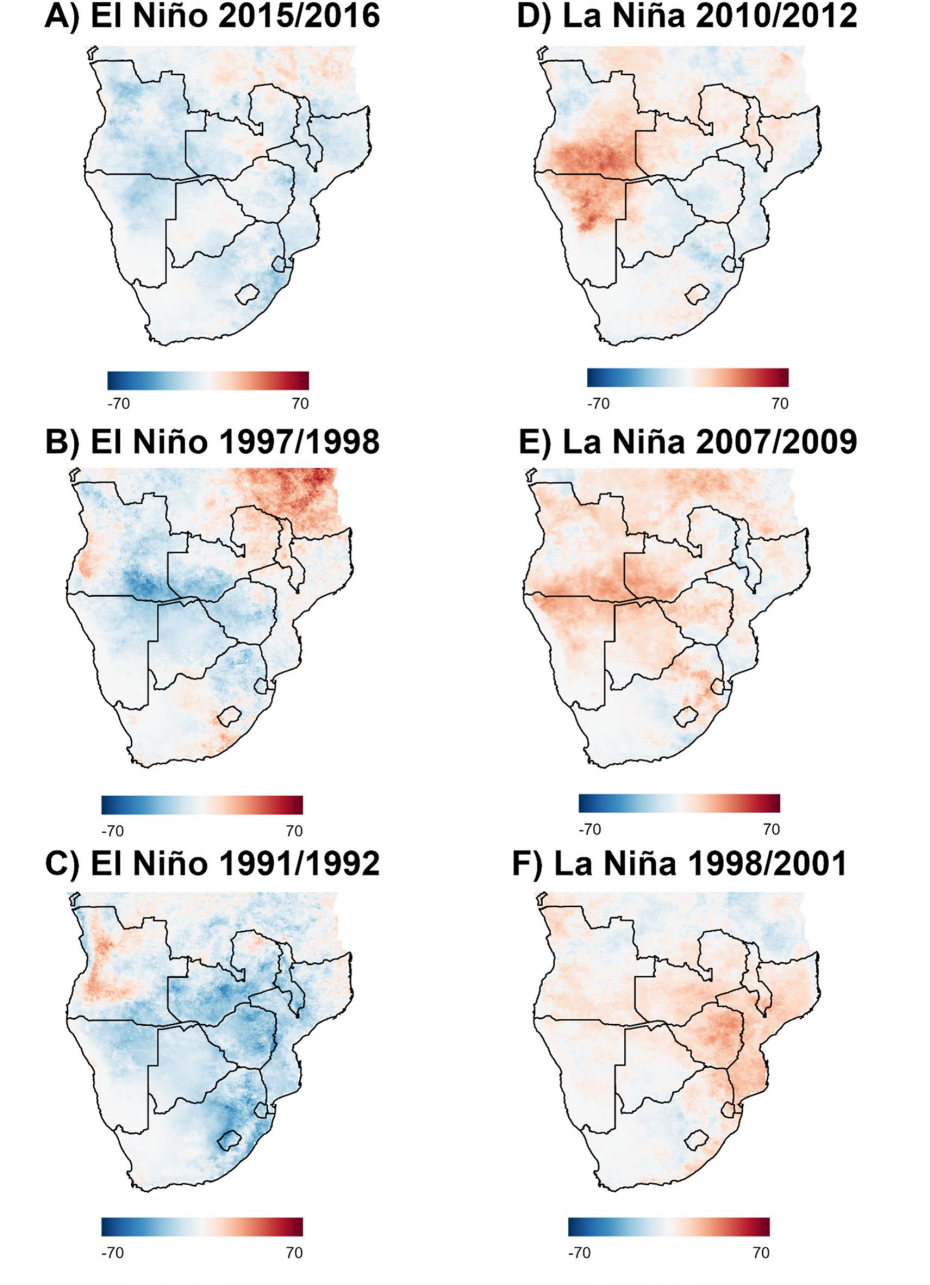
Choropleth map of rainy season *Q*_*0*_ anomalies relative to neutral conditions across the three strongest El Niño and La Niña events between 1987 and 2016 across southern Africa. Countries included in the analysis include Angola, Namibia, Botswana, Zambia, Zimbabwe, Eswatini, Mozambique, Malawi, South Africa and Lesotho. A southern portion of the Democratic Republic of the Congo is also visible, in addition to a southern portion of Tanzania.

To visualise the data geospatially, Fig. 7 (A-C) displays the geospatial distribution of anomalous *Q*_*0*_ values during three of the most significant El Niño and La Niña events across the typical rainy season in southern Africa. Comparison of A-C shows that *Q*_*0*_ is negatively modulated overall by El Niño in southern Africa, with particularly significant reductions occurring across southeastern Angola, southwestern Zambia, Zimbabwe, northern and eastern parts of South Africa and northeastern Namibia. Positive anomalies were present across all three events to varying degrees and across different parts of southern Africa. For example, although Malawi experienced moderate to strong anomalies in the 1991/1992 event, during the 1997/1998 event *Q*_*0*_ anomalies were positive. During the 1991/1992 and 1997/1998 events, large swathes of Angola experienced positive *Q*_*0*_ anomalies, although this was not the case during the 2015/2016 El Niño. Although the majority of anomalies were negative, indicating a reduction in transmission, there were pockets across the region where *Q*_*0*_ anomalies were absent across all three events. The arid and semiarid subtropical regions across Namibia’s southern and eastern regions, South Africa’s western region, and the southern tip of Botswana display negligible *Q*_*0*_ anomalies across all three events.

*Q*_*0*_ anomalies across the sampled La Niña events (2010–2012, 2007–2009, and 1998–2001) were generally characterised by widespread positive anomalies relative to neutral conditions, although greater heterogeneity in relation to the direction of change was observed compared to the sampled El Niño events. Particularly pronounced positive anomalies, or increases in transmission potential, were observed over southern Angola, northern Namibia and western Zambia for the 2010–2012 event (Fig. 7D), although mild negative anomalies occurred over northwestern Angola, Mozambique and northeastern South Africa. While less pronounced compared to the 2010–2012 event, Fig. 7E (2007–2009) shows positive *Q*_*0*_ anomalies occurred over southern Angola, northern Namibia and eastern Zambia. Eastern southern Africa experienced more pronounced positive *Q*_*0*_ anomalies than the western part of the region during the 1998–2001 La Niña event (Fig. 7F), whereas the 2010–2012 and 2007–2009 events exhibited the opposite pattern, reflective of stronger anomalies over the west of the region. Compared to the other two La Niña events (Fig. 7D and E), the 1998–2001 event (Fig. 7F) displays predominate increases in *Q*_*0*_ across eastern southern Africa rather than the western areas, with detected anomalies principally centred around Zimbabwe and Mozambique, in addition to southern areas of Malawi and Zambia. Decreases were observed in central areas of South Africa and northeastern Angola.

## Discussion

Despite the prominent effect of precipitation and temperature on *H. contortus* transmission and pathogenesis, alongside the prominent impact of ENSO on global climate variability and its consequences for agriculture in southern Africa, there is a dearth of literature which assesses the impact of ENSO on the transmission potential of *H. contortus*. This parasite imposes significant challenges to the health of small ruminants, important assets that play many roles for their owners, such as attenuating the negative impacts of crop failure on livelihoods. This deficit impedes an integrated understanding of the effects of ENSO on rural livelihoods and food security in southern Africa. The current study aimed to fill this vital gap by using the *Q*_*0*_ model to assess the direction and magnitude of ENSO’s effects on *H. contortus* transmission dynamics across AEZs in southern Africa.

### El Niño implications

Mean *Q*_*0*_ was largely unaffected by El Niño across the pooled analysis with exception to both tropical and subtropical cool-arid AEZs, the warm-subhumid subtropical AEZ and the warm-semiarid subtropical AEZ. However, this study suggests El Niño can significantly decrease *H. contortus* transmission potential during stronger El Niño events, with widespread medium-to-large effects observed in the case of the 1991/1992 and 2015/2016 events. This result is explained by significantly reduced precipitation during El Niño; such conditions constrain *H. contortus* infectivity through reduced development success during its free-living stages. However, without treatment existent infections will remain, the pathophysiological implications of which may be significant on small ruminant health if nutritional availability is likewise depressed.

El Niño has been shown across the literature to have negative effects on pasture productivity^1,8,57,58^. However, despite negative effects on vegetation, El Niño was not found to impact livestock in Botswana, likely due to supportive management interventions^8^. This emphasises the important role of supplemental feed in response plans. Enhanced wildfire incidences likely further amplify reductions in the availability of vegetation for grazing; Andela and van der Werf^59^ found ENSO contributed 51% to increased wildfire incidences in southern Africa, with results confirmed in a recent study by de Oliveira-Júnior, et al.^60^.

These El Niño-driven constraints on vegetation availability implicate negatively on animal nutrition in the absence of intervention. The combination between malnutrition and digestive parasitism often results in severe clinical signs and high mortality rates^61^, and as such nutrition is critical to optimise host responses to haemonchosis. This is especially important where *Q*_*0*_ is unaffected by El Niño, as infection pressure remains constant while nutritional availability may be depressed. Although infection pressure will likely reduce in areas where *Q*_*0*_ experiences notable declines, malnutrition will amplify the pathophysiological disturbances caused by concurrent infections. Walker et al. (2015), for instance, noted higher levels of anaemia in goats in Botswana at the end of the dry season, before *H. contortus* transmission increased, although goats that were treated with anthelmintics subsequently experienced greater improvement in anaemia scores. While treatments during dry periods including those exacerbated by El Niño might therefore be helpful, hostile climatic conditions are expected to reduce L3 availability on pasture and the persistence of drug-susceptible refugia^62,63^. This could result in higher parasite selection pressure and in turn risk accelerating the development of anthelmintic resistance. Since *H. contortus* has been shown to have high drug resistance potential^64,65^, this could negatively impact future options for its control. It is therefore important that treatments are applied responsibly, in line with sustainable parasite management strategies such as targeted selective treatment (TST).

### Implications of La Niña and the shift from El Niño

The results presented in section “Specific ENSO events” indicated that *Q*_*0*_ values tend to increase during La Niña compared to neutral phases across tropical AEZs. However, many results were not statistically significant despite many effect sizes being medium in magnitude, meaning we cannot be fully confident that the observed differences are not due to random variation. Effect sizes were often in or close to the medium effect size range though, implying that while the differences were not statistically significant, they were still meaningful in terms of practical relevance. The discrepancy between effect size and statistical significance could be explained by the limited number of La Niña data points, which reduces statistical power in contrast to the larger neutral phase dataset which allows for more robust statistical testing. Resultantly, the lack of statistical significance may not necessarily reflect the absence of a real effect but rather the limitations imposed by the smaller sample size which makes detecting statistical significance difficult even when medium effect sizes are detected.

Tropical AEZs were found to be comparatively more sensitive to *Q*_*0*_ perturbations during La Niña than subtropical zones. Statistically significant increases of transmission potential equivalent to a medium effect size were observed in the cool- and warm-semiarid AEZs, all tropical arid and semiarid zones (both warm and cool variants), along with the cool-subhumid zone. Contemporaneous climatic conditions in tropical zones mean baseline levels of endemic infectivity are already high due to the consistent presence of more favourable moisture conditions for *H. contortus* development year-round, as shown in the violin plots in Fig. 3. Transient climatic events such as La Niña further intensify humidity and moisture content, thus amplifying transmission potential.

Sudden transitions from El Niño to La Niña could have significant implications on small ruminant health. This study found consistent increases of *Q*_*0*_ equivalent to a medium effect size in comparisons between El Niño to La Niña, with statistically significant differences found over all tropical AEZs except for the cool-subhumid zone. In the subtropical AEZs, statistically significant increases of *Q*_*0*_ were observed solely within the warm-humid and warm-subhumid zones, likewise according to medium effect sizes.

As noted in section “El Niño implications”, the combination of malnutrition and parasitism leads to elevated levels of morbidity and mortality. This is particularly evident under tropical conditions on farms with alternating wet and dry seasons^61^, reflective of conditions prevalent during direct transitions from El Niño to La Niña. In the absence of intervention, poorly nourished animals, especially those that have been struggling to respond to existent infections over the El Niño period, are at high risk of mortality when *Q*_*0*_ swiftly increases during the wetter La Niña phase. *Haemonchus* has a notorious capacity for rapid population increases when climatic conditions are conducive to development, and burdens leading to widespread losses are known to occur quickly within short time periods following rainfall in warm climates^66^. Larvae have been found to have reached the upper stratum of grass after just 24 h following faecal deposition in warm and moist conditions, with the number of larvae peaking after seven days^67^. Given the propensity for eggs to rapidly hatch, develop to infective larvae and migrate to areas where risk of ingestion is high during conditions common in La Niña, regular checking of animals and quick responses to those displaying clinical signs of parasitism is very important to reduce associated losses and impacts on livelihoods and food security.

### Limitations and recommendations for future research

Spatiotemporal epidemiology studies are often subject to numerous limitations which may influence the validity of the results. To conduct time series analysis, data must be extracted from the study area using a particular methodology. Given the expanse of southern Africa, deciding a suitable methodology to segment the data to conduct time series analyses in a meaningful way is challenging. The methodology applied here was chosen as AEZs are categorised based on the existence of common climatic, geographic and ecological conditions which influence crop growth. It was assumed that, given the likewise importance of such factors for *H. contortus* epidemiology, segmenting the analysis using these zones would be a sensible approach. However, this remains a broad approach and transmission conditions are known to vary at the local level, especially rainfall. Therefore, whilst these results are useful for livestock producers, extension officers and veterinary services operating in these AEZs, they may not reflect exact experiences within particular AEZs. The best approach is to proactively conduct regular health checks and provide regular nutritional supplementation where possible, alongside targeted antiparasitic interventions (Ventura-Cordero et al., in review).

The climate data used to drive the model for this study also resulted in limitations which should be acknowledged. While both datasets are high resolution (0.05° or ~ 5 km), downloading and analysing the data was computationally demanding which reduced the scope for more comprehensive modelling and analysis. High-resolution data can provide more detailed insights, but they can also introduce noise if the data quality is inconsistent. CHIRPS precipitation data relies on station data, which is typically sparse and low quality in Africa^68^, especially in rural areas. The low availability and quality of station data may therefore affect the accuracy of *Q*_*0*_ estimates in this study.

Furthermore, this study analysed monthly *Q*_*0*_ data rather than daily. Use of a coarser temporal resolution meant intra-monthly variation was not captured by the analysis, which would have been particularly insightful to understand trends and implications of rapid climatic fluctuations, which are likely during ENSO transitions. Use of monthly data also resulted in a smaller number of data points within samples, especially for the individual event analyses, in turn reducing statistical power and increasing the uncertainty of the analysis. However, we maintain that use of monthly data enabled a reasonable approximation of transmission potential trends between El Niño, La Niña and neutral phases. It also simplified the analysis without losing overall trend information, offering a balanced approach that managed computational constraints. However, examining shorter time periods could be beneficial for identification of rapid changes in *Q*_*0*_, particularly during periods of heightened climatic variability, such as are common during ENSO transitions, which future work could focus on.

It is important to note that finer temporal resolution does not necessarily lead to more accurate model predictions. While *Q*_*0*_ can be calculated daily, it loses biological relevance if not averaged or calculated over a more appropriate timescale. For instance, if rain fails to occur on one day, *Q*_*0*_ for that day is zero, but rain occurring a few days before or after faecal deposition can still provide sufficient moisture for development. This is shown by^25,26^, who suggest a +/- four-day window is biologically appropriate, and thus a nine-day moving average suitably captures this, which the current model employs. As such, daily *Q*_*0*_ values could be misleading because many of the processes involved in transmission, including larval development and faecal drying which can take longer than this. Resulting time lags mean that the relevant time-period over which to average *Q*_*0*_ itself depends on climate and weather, and this warrants more research in order understand how daily data could be used for practical decision support.

A further note of caution is due as the parameters used within the *Q*_*0*_ model implemented in this study were based on European agroecological conditions^29,30^. As such, the results may not be entirely reflective of southern Africa. For example, the parameters dry matter intake, stocking density and standing biomass likely differ for extensive African compared to intensive European systems, and vary widely, although it was beyond the current study’s resources to calibrate and validate the model for African conditions, which themselves differ substantially across the continent. Stocking density was kept constant in this study to focus on the climatic drivers of transmission potential, and not on one of the potential confounders. To improve predictions, future research should calibrate and ground truth the *Q*_*0*_ model to be more representative of African systems. Work undertaken to develop the key parameters driving the model would need to be repeated in African conditions, and field data would need to be collected to validate this. Despite these potential sources of error, *Q*_*0*_ was found to correlate with faecal egg count and worm count data in select areas of Africa in a recent PhD thesis by Brown^69^.

Future work could also extend the model to assess the transmission potential of other gastrointestinal nematodes prevalent in Africa. For example, a model for *Trichostrongylus colubriformis* could be developed using parameters described in O’Connor, Walkden-Brown and Kahn^70^. However, *Q*_*0*_ is particularly apt for modelling *Haemonchus* because it is dominant across most of Africa, and because it has a high fecundity and fast life cycle, meaning positive weather can result in explosive population increases and disease outbreaks. For other parasites with lower biotic potential, other factors such as grazing management, animal movement and nutritional status become relatively more important, relative to climatic conditions.

While ENSO has been shown to affect *H. contortus* transmission potential in the current study, it is not currently known how climate change may impact *Q*_*0*_ in Africa. Future research should therefore aim to fill this pertinent gap, which would provide valuable insights in support of NGOs, governments and extension workers developing effective climate adaptation strategies for rural farmers facing the challenges of climate change across Africa.

### Implications for policy and practice

In cases where ENSO is active, particularly during transitions between phases, effective small ruminant health monitoring and responsible application of anthelmintic treatments is vital to manage haemonchosis, while safeguarding against anthelmintic resistance. Providing nutritional supplementation improves host responses to infection, especially when vegetation availability is low and thus animal nutrition constrained, such as during El Niño, or when climatic conditions are particularly conducive to transmission, such as across particularly rainy periods common in La Niña. Rotational grazing is a key preventative strategy to minimise production losses, and alongside providing drug treatments, impacts of haemonchosis can attenuated if an appropriate system is effectively implemented^66^. For farmers operating on communal grazing lands, who overwhelmingly tend to be resource-poor, rotational grazing will not be feasible. As such, alternative strategies focusing on early identification of individual animals struggling with infections will enable early intervention, providing a better chance of more desirable outcomes. Furthermore, regular health monitoring of animals, nutritional supplementation, and application of drugs using targeted selective treatment (TST) are vital strategies to avoid cascading negative impacts, especially when combined. However, provision of formal nutritional supplementation and access to parasite diagnostics or health interventions is not often possible for resource-poor farmers, who hold 75% of southern Africa’s livestock population according to the Southern Africa Development Community (SADC)^71^.

Alternative low-cost solutions are therefore vital. The Five Point Check (FPC) is a low-cost tool that helps farmers to estimate the impact of GIN infections on their herds, allowing for the application of anthelmintics via TST^72^. Application of TST was shown in Botswana to benefit herd health just as much as whole herd treatments, costing 83% less while safeguarding against resistance^19^. In the absence of drugs, ethnobotanical, nutrition-rich plants containing plant secondary metabolites (PSMs) such as condensed tannins can improve host response to infections and represent indigenous animal health solutions resource-poor farmers utilise to respond to disease^61,73–75^. TST has more recently been applied using combinations of drugs and ethnobotanical plants in Malawi, showing beneficial effects on host responses to infection (Ventura-Cordero et al., in review). Therefore, dissemination of the FPC and TST is likely to bolster smallholders’ ability to identify and manage infections. However, inadequate drug availability and veterinary support in rural areas of Africa may mean resource-poor farmers are reliant on ethnobotanical solutions, which have their limits in terms of efficacy.

While climatic factors are unlikely to affect the availability of ethnobotanical plants for TST during La Niña due to the typically wetter conditions, a major issue with farmers relying on these during El Niño is that climatic conditions may reduce their availability. As part of preparedness plans, researchers could work with NGOs and governments to develop and build capacity amongst farmers to produce homemade bioactive supplements. The research would develop a recipe using, for example, locally available plants containing PSMs, seeds and crop residues to produce a dried high protein supplement that can easily be made by farmers. Trials would be needed to assess the effectiveness of the supplement on animal productivity, responses to infections and faecal egg counts, and should include recipe substitutions in case a particular ingredient is not available. These supplements could be safely stored and used when needed. Once an effective recipe has been developed, NGOs and government livestock development departments could disseminate the method of production, allowing farmers to produce their own supplements. It is highly recommended that the intervention, following appropriate training of farmers, simultaneously distributes FAMACHA cards and teaches farmers and extension workers how to undertake the FPC to improve their capacity to identify animals struggling with infections and allow for TST.

The present study showed many changes in *Q*_*0*_ are statistically significant across ENSO events, which will present challenges for small ruminant farmers, many of whom operate in mixed farming systems where crops will likewise be affected. Highly variable and challenging weather conditions resulting from ENSO in southern Africa are known to affect growing conditions and yields for vegetation and key crops^57^, and while yields can be higher during La Niña than average yields in neutral conditions, yield decreases during El Niño are usually larger than corresponding increases resulting from La Niña^1^. This suggests that during La Niña, practitioners should prioritise helping farmers manage higher levels of *H. contortus* infections resulting from enhanced precipitation.

Goats are frequently employed as emergency assets that can be sold during challenging periods^11,73^, so it is important that their health and productivity is optimised. However, the statistically significant changes observed in the current study highlight the potential for sudden increases in *H. contortus* transmission potential, which means farmers are likely to experience sudden and impactful levels of heightened morbidity and mortality among herds. Being aware of and characterising this variation can assist practitioners to proactively develop ENSO preparedness plans to ensure small ruminants play the important roles which farmers depend upon. Since impacts on small ruminants will be non-independent of those on crops, there is considerable potential to coordinate dissemination efforts and logistical support for farmers across both areas. The 2016 El Niño event led to a 9.3 million tonne crop production shortfall and more than 643,000 livestock deaths in southern Africa^76^. FAO supported livestock farmers across the region with livestock survival feed, destocking and restocking with small ruminants, animal health interventions (which in the Malawi case included deworming for goats), rehabilitation of water points and livestock vaccination and restocking using small ruminants^76,77^. However, research assessing the effectiveness of these interventions was not found, indicating a requirement for future research.

## Conclusion

This study set out to assess the impact of ENSO on *H. contortus* transmission potential across AEZs in southern Africa during the region’s typical rainy season. Results from the pooled event analysis indicate that El Niño does not generally have a significant impact on *Q*_*0*_ across subtropical AEZs, except for the warm-semiarid subtropical zone where a significant decrease in *Q*_*0*_ was observed, equivalent to a medium effect size. However, specific strong El Niño events were found to be significantly associated with substantial reductions in *H. contortus* transmission potential, as indicated by medium-to-large effect sizes, which were observed in 50% of all sampled AEZs. While tropical AEZs typically demonstrated lower sensitivity to El Niño compared to subtropical equivalents, in the case of certain strong events significant reductions in *Q*_*0*_ were observed within some tropical zones.

La Niña did not generally have a significant impact on *H. contortus* transmission potential compared to neutral *Q*_*0*_ values in subtropical AEZs, across both pooled and individual event analysis, except for the case of the 2007–2009 episode. During this event, *Q*_*0*_ increased across the warm-semiarid AEZ by a medium effect size magnitude. However, it was found that tropical AEZs were comparatively more sensitive to *Q*_*0*_ disturbances during La Niña, with *Q*_*0*_ significantly increasing according to a medium effect size in the cool- and warm-semiarid tropical AEZs. All variations of tropical arid and semiarid zones (both warm and cool), as well as the cool-subhumid zone, displayed at least one instance of increased transmission potential according to a medium effect size.

Significantly, large increases in *Q*_*0*_ during shifts from El Niño to La Niña were detected by the analysis, with statistically significant medium effects observed across all tropical AEZs except for the cool-subhumid zone. In subtropical AEZs, notable increases in *Q*_*0*_ were detected within the warm-humid and warm-subhumid zones, also demonstrating medium effect sizes. Farmers and extension officers should be particularly vigilant with undertaking regular herd health checks when El Niño shifts directly to La Niña, as sudden, strong increases in transmission potential are likely to occur, risking enhanced morbidity and mortality across herds.

It is recommended that during ENSO events, small ruminant farmers undertake particularly regular inspections of their herds, undertaking regular health examinations, supplementing nutrition where possible and only treat animals showing signs of infection using anthelmintics and locally available ethnobotanical plants. Given the inability of resource-poor farmers to access traditional veterinary diagnostic services and tools, low-cost methods such as the FPC are advised to be disseminated by NGOs and governments to allow for mitigative measures to be implemented.

## Electronic supplementary material

Below is the link to the electronic supplementary material.

Supplementary Material 1

## Data Availability

Data is available upon request from the corresponding author (Jonathan H. I. Tinsley, jtinsley03@qub.ac.uk).

## References

[1] Sazib, N., Mladenova, E. & Bolten, J. D. Assessing the impact of ENSO on agriculture over Africa using Earth observation data. *Front. Sustainable Food Syst.* **4**10.3389/fsufs.2020.509914 (2020).

[2] Lin, J. & Qian, T. A. New picture of the global impacts of El Nino-Southern Oscillation. *Sci. Rep.* **9**, 17543. 10.1038/s41598-019-54090-5 (2019). 31772238 10.1038/s41598-019-54090-5 PMC6879734

[3] Hoell, A., Gaughan, A. E., Magadzire, T. & Harrison, L. The modulation of daily Southern Africa precipitation by El Niño–Southern Oscillation across the summertime wet season. *J. Clim.* **34**, 1115–1134. 10.1175/JCLI-D-20-0379.1 (2021).

[4] Torres-Alavez, J. A., Giorgi, F., Kucharski, F. & Coppola, E. Castro-García, L. ENSO teleconnections in an ensemble of CORDEX-CORE regional simulations. *Clim. Dyn.* **57**, 1445–1461. 10.1007/s00382-020-05594-8 (2021).

[5] Mugiyo, H. et al. El Niño’s Effects on Southern African Agriculture in 2023/24 and Anticipatory Action Strategies to Reduce the Impacts in Zimbabwe. *Atmosphere* **14** (2023).

[6] Bradshaw, C. D. et al. Unprecedented climate extremes in South Africa and implications for maize production. *Environ. Res. Lett.* **17**, 13. 10.1088/1748-9326/ac816d (2022).

[7] Sivakumar, M. V. K., Das, H. P. & Brunini, O. Impacts of present and future climate variability and change on agriculture and forestry in the arid and semi-arid tropics. *Clim. Change*. **70**, 31–72. 10.1007/s10584-005-5937-9 (2005).

[8] Stige, L. C. et al. The effect of climate variation on agro-pastoral production in Africa. *Proceedings of the National Academy of Sciences* 103, 3049–3053 (2006). 10.1073/pnas.0600057103 10.1073/pnas.0600057103 PMC1413945 16492727

[9] Phillips, J. G., Cane, M. A. & Rosenzweig, C. ENSO, seasonal rainfall patterns and simulated maize yield variability in Zimbabwe. *Agric. Meteorol.* **90**, 39–50. 10.1016/s0168-1923(97)00095-6 (1998).

[10] Acosta, A., Nicolli, F. & Karfakis, P. Coping with climate shocks: the complex role of livestock portfolios. *World Dev.* **146**, 105546. 10.1016/j.worlddev.2021.105546 (2021). https://doi.org:.

[11] Kaumbata, W. et al. Tangible and intangible benefits of local goats rearing in smallholder farms in Malawi. *Small Ruminant Res.* **187**, 106095. 10.1016/j.smallrumres.2020.106095 (2020). https://doi.org:.

[12] Phiri, E. The Effectiveness of the Goat Value Chain on Poverty Reduction among Smallholder Farming Households in Shurugwi District’s Ward 9. (2015).

[13] Gracinda Andre, M., Carina, V. & Alcides, S. in *Goat Science* (ed Kukovics Sándor) Ch. 7IntechOpen, (2021).

[14] Bett, B. et al. Effects of climate change on the occurrence and distribution of livestock diseases. *Prev. Vet. Med.* **137**, 119–129. 10.1016/j.prevetmed.2016.11.019 (2017). 28040271 10.1016/j.prevetmed.2016.11.019

[15] Oyas, H. et al. Enhanced surveillance for rift Valley fever in livestock during El Nino rains and threat of RVF outbreak, Kenya, 2015–2016. *Plos Negl. Trop. Dis.* **12**, 15. 10.1371/journal.pntd.0006353 (2018). 10.1371/journal.pntd.0006353 PMC5919633 29698487

[16] Redding, D. W., Tiedt, S., Lo Iacono, G., Bett, B. & Jones, K. E. Spatial, seasonal and Climatic predictive models of rift Valley fever disease across Africa. *Philos. Trans. R Soc. B-Biol Sci.* **372**, 9. 10.1098/rstb.2016.0165 (2017). 10.1098/rstb.2016.0165 PMC5468690 28584173

[17] Fandamu, P., Duchateau, L., Speybroeck, N., Mulumba, M. & Berkvens, D. East Coast fever and multiple El Nino Southern Oscillation ranks. *Vet. Parasitol.* **135**, 147–152. 10.1016/j.vetpar.2005.09.008 (2006). 16213095 10.1016/j.vetpar.2005.09.008

[18] Perry, B. D., Randolph, R. F., McDermott, J. J., Sones, K. R. & Thornton, P. K. Investing in animal health research to alleviate poverty. *International Livest. Res. Inst. (ILRI)* (2002).

[19] Walker, J. G. et al. Mixed methods evaluation of targeted selective anthelmintic treatment by resource-poor smallholder goat farmers in Botswana. *Vet. Parasitol.* **214**, 80–88. 10.1016/j.vetpar.2015.10.006 (2015). 26493540 10.1016/j.vetpar.2015.10.006 PMC4671485

[20] Sargison, N. The critical importance of planned small ruminant livestock health and production in addressing global challenges surrounding food production and poverty alleviation. *N. Z. Vet. J.* **68**, 136–144. 10.1080/00480169.2020.1719373 (2020). 31968203 10.1080/00480169.2020.1719373

[21] Besier, R. B., Kahn, L. P., Sargison, N. D. & Van Wyk, J. A. in *Advances in Parasitology* Vol. 93 (eds Robin B. Gasser & Georg Von Samson-Himmelstjerna) 95–143Academic Press, (2016).

[22] Van Dijk, J. & Morgan, E. R. The influence of water on the migration of infective trichostrongyloid larvae onto grass. *Parasitology* **138**, 780–788. 10.1017/s0031182011000308 (2011). 24650934 10.1017/S0031182011000308

[23] O’Connor, L. J., Walkden-Brown, S. W. & Kahn, L. P. Ecology of the free-living stages of major trichostrongylid parasites of sheep. *Vet. Parasitol.* **142**, 1–15. 10.1016/j.vetpar.2006.08.035 (2006). 17011129 10.1016/j.vetpar.2006.08.035

[24] O’Connor, L. J., Kahn, L. P. & Walkden-Brown, S. W. The effects of amount, timing and distribution of simulated rainfall on the development of Haemonchus contortus to the infective larval stage. *Vet. Parasitol.* **146**, 90–101. 10.1016/j.vetpar.2007.02.002 (2007). 17398009 10.1016/j.vetpar.2007.02.002

[25] Khadijah, S., Kahn, L. P., Walkden-Brown, S. W., Bailey, J. N. & Bowers, S. F. Soil moisture influences the development of Haemonchus contortus and trichostrongylus colubriformis to third stage larvae. *Vet. Parasitol.* **196**, 161–171. 10.1016/j.vetpar.2013.01.010 (2013). 23398986 10.1016/j.vetpar.2013.01.010

[26] Khadijah, S., Kahn, L. P., Walkden-Brown, S. W., Bailey, J. N. & Bowers, S. F. Soil moisture modulates the effects of the timing and amount of rainfall on faecal moisture and development of Haemonchus contortus and trichostrongylus colubriformis to infective third stage larvae. *Vet. Parasitol.* **196**, 347–357. 10.1016/j.vetpar.2013.03.034 (2013). 23632251 10.1016/j.vetpar.2013.03.034

[27] Wang, T. et al. Microclimate has a greater influence than macroclimate on the availability of infective Haemonchus contortus larvae on herbage in a warmed temperate environment. *Agric. Ecosyst. Environ.* **265**, 31–36. 10.1016/j.agee.2018.05.029 (2018).

[28] Wang, T., Van Wyk, J. A., Morrison, A. & Morgan, E. R. Moisture requirements for the migration of Haemonchus contortus third stage larvae out of faeces. *Vet. Parasitol.* **204**, 258–264. 10.1016/j.vetpar.2014.05.014 (2014). 24893698 10.1016/j.vetpar.2014.05.014

[29] Rose, H. et al. Climate-driven changes to the spatio‐temporal distribution of the parasitic nematode, Haemonchus contortus, in sheep in Europe. *Glob. Change Biol.* **22**, 1271–1285. 10.1111/gcb.13132 (2016). 10.1111/gcb.13132 26482823

[30] Bolajoko, M. B. et al. The basic reproduction quotient (Q0) as a potential Spatial predictor of the seasonality of ovine haemonchosis. *Geospat. Health*. **9**, 333–350. 10.4081/gh.2015.356 (2015). 25826315 10.4081/gh.2015.356

[31] Walker, J. G., Evans, K. E., Vineer, R., van Wyk, H., Morgan, E. R. & J. A. & Prediction and Attenuation of seasonal spillover of parasites between wild and domestic ungulates in an arid mixed-use system. *J. Appl. Ecol.* **55**, 1976–1986. 10.1111/1365-2664.13083 (2018). 30008482 10.1111/1365-2664.13083 PMC6032883

[32] Heesterbeek, J. & Roberts, M. Threshold quantities for helminth infections. *J. Math. Biol.* **33**, 415–434 (1995). 7714416 10.1007/BF00176380

[33] Diekmann, O., Heesterbeek, J. A. & Metz, J. A. On the definition and the computation of the basic reproduction ratio R0 in models for infectious diseases in heterogeneous populations. *J. Math. Biol.* **28**, 365–382. 10.1007/bf00178324 (1990). 2117040 10.1007/BF00178324

[34] Roberts, M. G. & Heesterbeek, J. A. P. Model-consistent Estimation of the basic reproduction number from the incidence of an emerging infection. *J. Math. Biol.* **55**, 803–816. 10.1007/s00285-007-0112-8 (2007). 17684743 10.1007/s00285-007-0112-8 PMC2782110

[35] Coyne, M. J. & Smith, G. The mortality and fecundity of Haemonchus contortus in parasite-naive and parasite-exposed sheep following single experimental infections. *Int. J. Parasitol.* **22**, 315–325. 10.1016/s0020-7519(05)80009-8 (1992). 1639567 10.1016/s0020-7519(05)80009-8

[36] Barger, I. A. & Le Jambre, L. F. Regulation of Haemonchus contortus populations in sheep: mortality of established worms. *Int. J. Parasitol.* **18**, 269–273. 10.1016/0020-7519(88)90067-7 (1988). 3372130 10.1016/0020-7519(88)90067-7

[37] O’Connor, L. J., Kahn, L. P. & Walkden-Brown, S. W. Interaction between the effects of evaporation rate and amount of simulated rainfall on development of the free-living stages of Haemonchus contortus. *Vet. Parasitol.* **155**, 223–234. 10.1016/j.vetpar.2008.05.010 (2008). 18586404 10.1016/j.vetpar.2008.05.010

[38] Khadijah, S., Kahn, L. P., Walkden-Brown, S. W., Bailey, J. N. & Bowers, S. F. Effect of simulated rainfall timing on faecal moisture and development of Haemonchus contortus and trichostrongylus colubriformis eggs to infective larvae. *Vet. Parasitol.* **192**, 199–210. 10.1016/j.vetpar.2012.10.015 (2013). 23142178 10.1016/j.vetpar.2012.10.015

[39] Rose, H., Wang, T., Van Dijk, J., Morgan, E. R. & GLOWORM-FL: A simulation model of the effects of climate and climate change on the free-living stages of gastro-intestinal nematode parasites of ruminants. *Ecol. Model.* **297**, 232–245. 10.1016/j.ecolmodel.2014.11.033 (2015).

[40] Callinan, A. P. & Westcott, J. M. Vertical distribution of trichostrongylid larvae on herbage and in soil. *Int. J. Parasitol.* **16**, 241–244. 10.1016/0020-7519(86)90050-0 (1986). 3744667 10.1016/0020-7519(86)90050-0

[41] Barger, I. A., Jambre, L., Georgi, L. F., Davies, H. I. & J. R. & Regulation of Haemonchus contortus populations in sheep exposed to continuous infection. *Int. J. Parasitol.* **15**, 529–533. 10.1016/0020-7519(85)90049-9 (1985). 4066146 10.1016/0020-7519(85)90049-9

[42] Kao, R. R., Leathwick, D. M., Roberts, M. G. & Sutherland, I. A. Nematode parasites of sheep: a survey of epidemiological parameters and their application in a simple model. *Parasitology* **121** (Pt 1), 85–103. 10.1017/s0031182099006095 (2000). 11085228 10.1017/s0031182099006095

[43] Leathwick, D. M., Barlow, N. D. & Vlassoff, A. A model for nematodiasis in new Zealand lambs. *Int. J. Parasitol.* **22**, 789–799. 10.1016/0020-7519(92)90129-9 (1992). https://doi.org:. 1428512 10.1016/0020-7519(92)90129-9

[44] Funk, C. et al. The climate hazards infrared precipitation with stations—a new environmental record for monitoring extremes. *Sci. Data*. **2**, 150066. 10.1038/sdata.2015.66 (2015). 26646728 10.1038/sdata.2015.66 PMC4672685

[45] Hamon, W. R. Estimating potential evapotranspiration. *J. Hydraulics Div.* **87**, 107–120. 10.1061/JYCEAJ.0000599 (1961).

[46] Verdin, A. et al. Development and validation of the CHIRTS-daily quasi-global high-resolution daily temperature data set. *Sci. Data*. **7**, 303. 10.1038/s41597-020-00643-7 (2020). 32929097 10.1038/s41597-020-00643-7 PMC7490712

[47] Funk, C. et al. A High-Resolution 1983–2016 Tmax climate data record based on infrared temperatures and stations by the climate hazard center. *J. Clim.* **32**, 5639–5658. 10.1175/JCLI-D-18-0698.1 (2019).

[48] Funk, C. C. et al. Reston, VA,. A quasi-global precipitation time series for drought monitoring. Report No. 832, 12 (2014).

[49] CDO User Guide (2.3.0). Zenodo, (2023).

[50] ArcGIS & Pro *3.2.0* (Redlands, CA, 2023).

[51] Sebastian, K. (ed) HarvestChoice) (Harvard Dataverse, (2009).

[52] Arsenopoulos, K. V., Fthenakis, G. C., Katsarou, E. I. & Papadopoulos, E. Haemonchosis: A challenging parasitic infection of sheep and goats. *Animals* **11**, 363 (2021). 33535656 10.3390/ani11020363 PMC7912824

[53] Hoberg, E. P. & Zarlenga, D. S. in *Advances in Parasitology* Vol. 93 (eds Robin B. Gasser & Georg Von Samson-Himmelstjerna) 1–30Academic Press, (2016).

[54] RStudio & Boston Integrated Development Environment for R. (MA., (2022).

[55] Cohen, J. *Statistical Power Analysis for the Behavioral Sciences* (Routledge, 2013).

[56] NOAA. *Past Events*, https://psl.noaa.gov/enso/past_events.html (n.d.).

[57] Hao, Y., Hao, Z., Feng, S., Zhang, X. & Hao, F. Response of vegetation to El Niño-Southern Oscillation (ENSO) via compound dry and hot events in Southern Africa. *Glob. Planet Change*. **195**, 103358. 10.1016/j.gloplacha.2020.103358 (2020).

[58] Anyamba, A. & Eastman, J. R. Interannual variability of NDVI over Africa and its relation to El Niño/Southern Oscillation. *Int. J. Remote Sens.* **17**, 2533–2548. 10.1080/01431169608949091 (1996).

[59] Andela, N. & van der Werf, G. R. Recent trends in African fires driven by cropland expansion and El Niño to La Niña transition. *Nat. Clim. Change*. **4**, 791–795. 10.1038/nclimate2313 (2014).

[60] de Oliveira-Júnior, J. F. et al. Impact of the El Niño on fire dynamics on the African continent. *Earth Syst. Environ.* **8**, 45–61. 10.1007/s41748-023-00363-z (2024).

[61] Hoste, H. et al. Academic Press,. in *Advances in Parasitology* Vol. 93 (eds Robin B. Gasser & Georg Von Samson-Himmelstjerna) 239–351 (2016).

[62] van Wyk, J. A. Refugia–overlooked as perhaps the most potent factor concerning the development of anthelmintic resistance. *Onderstepoort J. Vet. Res.* **68**, 55–67 (2001). 11403431

[63] Hodgkinson, J. E. et al. Refugia and anthelmintic resistance: concepts and challenges. *Int. J. Parasitol. Drugs Drug Resist.* **10**, 51–57. 10.1016/j.ijpddr.2019.05.001 (2019). 31125837 10.1016/j.ijpddr.2019.05.001 PMC6531808

[64] Knapp-Lawitzke, F., von Samson-Himmelstjerna, G. & Demeler, J. Elevated temperatures and long drought periods have a negative impact on survival and fitness of strongylid third stage larvae. *Int. J. Parasitol.* **46**, 229–237. 10.1016/j.ijpara.2015.10.006 (2016). 26828893 10.1016/j.ijpara.2015.10.006

[65] Kotze, A. C. & Prichard, R. K. in *Advances in Parasitology* Vol. 93 (eds Robin B. Gasser & Georg Von Samson-Himmelstjerna) 397–428Academic Press, (2016).

[66] Besier, R. B., Kahn, L. P., Sargison, N. D. & Van Wyk, J. A. in *Advances in Parasitology* Vol. 93 (eds Robin B. Gasser & Georg Von Samson-Himmelstjerna) 181–238Academic Press, (2016).

[67] Santos, M. C., Silva, B. F. & Amarante, A. F. T. Environmental factors influencing the transmission of Haemonchus contortus. *Vet. Parasitol.* **188**, 277–284. 10.1016/j.vetpar.2012.03.056 (2012). 22521972 10.1016/j.vetpar.2012.03.056

[68] Dinku, T. in *Extreme Hydrology and Climate Variability* (eds Assefa M. Melesse, Wossenu Abtew, & Gabriel Senay) 71–80Elsevier, (2019).

[69] Brown, T. *Managing biosecurity risks from drug-resistant parasites and other diseases in deer* Doctor of Philosophy thesis, Queen’s University Belfast, (2023).

[70] O’Connor, L. J., Walkden-Brown, S. W. & Kahn, L. P. Ecology of the free-living stages of major trichostrongylid parasites of sheep. *Vet. Parasitol.* **142**, 1–15. 10.1016/j.vetpar.2006.08.035 (2006). 17011129 10.1016/j.vetpar.2006.08.035

[71] SADC. *Livestock production*, https://www.sadc.int/pillars/livestock-production (n.d.).

[72] Bath, G. F. & Van Wyk, J. A. The five point check© for targeted selective treatment of internal parasites in small ruminants. *Small Ruminant Res.* **86**, 6–13. 10.1016/j.smallrumres.2009.09.009 (2009).

[73] Airs, P. M. et al. Goat health and management for improved smallholders’ livelihoods in central Malawi – A socioeconomic analysis of rural households. *Small Ruminant Res.* **229**10.1016/j.smallrumres.2023.107114 (2023).

[74] French, K. E. Plant-Based solutions to global livestock anthelmintic resistance. *Ethnobiol. Lett.* **9**10.14237/ebl.9.2.2018.980 (2018).

[75] Oliveira Santos, F., Morais Cerqueira, P., Branco, A., José Moreira Batatinha, A., Borges Botura, M. & M. & Anthelmintic activity of plants against Gastrointestinal nematodes of goats: a review. *Parasitology* **146**, 1233–1246. 10.1017/S0031182019000672 (2019). 31104640 10.1017/S0031182019000672

[76] FAO. *El Niño: Preparedness and Response*, (2016). https://www.fao.org/fileadmin/user_upload/emergencies/docs/FAOElNinoSitRep_APRIL2016b.pdf

[77] FAO. *Southern Africa*, (2016). https://www.fao.org/fileadmin/user_upload/emergencies/docs/Southern%20Africa%20Situation%20Report_September%202016.pdf

