# Supplementary material for: El Niño and La Niña differentially drive transmission dynamics of the small ruminant parasite Haemonchus contortus across agroecological zones in Southern Africa
Source: Sci Rep. 2025 Jul 1;15:22045. doi: 10.1038/s41598-025-03156-8 (PMC12216351; doi:10.1038/s41598-025-03156-8)
Supplement: Supplementary file 1 — Supplementary Material 1 [file 41598_2025_3156_MOESM1_ESM.docx]

Supplementary Materials

| **Table S1**: Impact of El Niño phase changes on *H. contortus* transmission potential during the typical rainy season (November-April) across the 14 different agroecological zones of southern Africa. Notable results are highlighted in bold text. | | | | | | | | | | | | | | |
| --- | --- | --- | --- | --- | --- | --- | --- | --- | --- | --- | --- | --- | --- | --- |
|  | **Subtropical AEZs** | | | | | | | **Tropical AEZs** | | | | | | |
|  | **Cool-arid** | **Cool-semiarid** | **Warm-humid** | **Warm-subhumid** | **Warm-semiarid** | **Warm-arid** | **Cool-subhumid** | **Warm-arid** | **Warm-semiarid** | **Warm-subhumid** | **Warm-humid** | **Cool-arid** | **Cool-semiarid** | **Cool-subhumid** |
| **Neutral-El Niño** | | | | | | | | | | | | | | |
| **T-stat** | 2.722 | 1.877 | 1.565 | 2.415 | 3.384 | 1.711 | 0.021 | 1.416 | 121.43 | 0.240 | 1.099 | 2.5 | -0.738 | -1.607 |
| **Degrees of freedom** | 121.780 | 116.010 | 113.310 | 118.06 | 121.700 | 89.565 | 111.550 | 113.650 | 0.798 | 121.910 | 120.580 | 121.790 | 121.130 | 121.260 |
| **P-value** | **0.007*** | 0.063 | 0.120 | **0.017*** | **0.0009**** | 0.090 | 0.984 | 0.160 | 0.256 | 0.810 | 0.274 | **0.014*** | 0.462 | 0.110 |
| **95^th^ % confidence interval** | 0.856 | -0.356 | -2.608 | 1.500 | 2.687 | -0.095 | -9.736 | -1.075 | -7.472 | -5.955 | -4.145 | 0.965 | -13.194 | -23.899 |
|  | 5.421 | 13.657 | 22.239 | 15.173 | 10.263 | 1.271 | 9.941 | 6.460 | 9.692 | 7.603 | 14.484 | 8.301 | 6.030 | 2.482 |
| **Sample estimates: mean of x (N), mean of y (EN)** | 5.185 | 29.225 | 62.347 | 41.634 | 16.880 | 1.133 | 51.713 | 9.445 | 45.008 | 84.160 | 132.568 | 10.407 | 68.066 | 113.593 |
|  | 2.046 | 22.579 | 52.532 | 33.297 | 10.405 | 0.544 | 51.611 | 6.752 | 43.898 | 83.337 | 127.399 | 5.774 | 71.648 | 124.302 |
| **Percentage change** | -61% | -23% | -16% | -20% | -38% | -52% | 0% | -29% | -2% | -1% | -4% | -45% | 5% | 9% |
| **Effect size** | 0.468 | 0.335 | 0.282 | 0.429 | 0.581 | 0.274 | 0.004 | 0.254 | 0.044 | 0.041 | 0.193 | 0.430 | -0.129 | -0.277 |
| **Magnitude** | Small | Small | Small | Small | **Medium** | Small | Negligible | Small | Negligible | Negligible | Negligible | Small | Negligible | Small |
| **Neutral-La Niña** | | | | | | | | | | | | | | |
| **T-stat** | 1.482 | 1.047 | -1.468 | -0.776 | 1.047 | 1.374 | -2.046 | -2.194 | -3.030 | -2.368 | -1.418 | -1.711 | -3.348 | -2.810 |
| **Degrees of freedom** | 124.930 | 124.300 | 119.630 | 118.810 | 124.300 | 114.150 | 117.360 | 87.514 | 112.410 | 123.400 | 117.090 | 85.094 | 122.510 | 123.600 |
| **P-value** | 0.141 | 0.2972 | 0.145 | 0.439 | 0.297 | 0.172 | **0.042*** | **0.031*** | **0.003*** | **0.019*** | 0.159 | 0.090 | **0.001*** | **0.006*** |
| **95^th^ % confidence interval** | -0.585 | -1.909 | -21.190 | -9.899 | -1.909 | -0.231 | -19.738 | -10.874 | -25.436 | -16.075 | -13.890 | -11.265 | -26.969 | -32.449 |
|  | 4.071 | 6.194 | 3.145 | 4.323 | 6.194 | 1.275 | -0.323 | -0.537 | -5.323 | -1.437 | 2.300 | 0.845 | -6.927 | -5.630 |
| **Sample estimates: mean of x (N), mean of y (LN)** | 5.185 | 16.880 | 62.347 | 41.634 | 16.880 | 1.133 | 51.714 | 9.444 | 45.008 | 84.160 | 132.568 | 10.407 | 68.066 | 113.593 |
|  | 3.344 | 14.738 | 71.369 | 44.422 | 14.738 | 0.610 | 61.744 | 15.150 | 60.387 | 92.916 | 138.363 | 15.617 | 85.014 | 132.633 |
| **Percentage change** | -36% | -13% | 14% | 7% | -13% | -46% | 19% | 60% | 34% | 10% | 4% | 50% | 25% | 17% |
| **Effect size** | 0.255 | -0.129 | -0.259 | -0.138 | 0.182 | 0.230 | -0.364 | -0.410 | -0.545 | -0.414 | -0.238 | -0.321 | -0.588 | -0.485 |
| **Magnitude** | Small | Negligible | Small | Negligible | Negligible | Small | Small | Small | **Medium** | Small | Small | Small | **Medium** | Small |
| **El Niño-La Niña** | | | | | | | | | | | | | | |
| **T-stat** | -1.312 | -2.3735 | -2.887 | -3.052 | -2.326 | -0.277 | -1.920 | -3.130 | -3.348 | -2.809 | -2.757 | -3.351 | -2.721 | -1.377 |
| **Degrees of freedom** | 106.960 | 105.640 | 106.560 | 106.780 | 105.370 | 89.945 | 106.730 | 91.650 | 100.250 | 104.960 | 94.046 | 75.262 | 106.650 | 107.950 |
| **P-value** | 0.192 | **0.0194*** | **0.0048*** | **0.003*** | 0.022 | 0.782 | 0.058 | **0.002*** | **0.001*** | **0.006*** | **0.007*** | **0.001*** | **0.008*** | 0.171 |
| **95^th^ % confidence interval** | -3.504 | -17.273 | -31.798 | -18.353 | -8.026 | -0.539 | -20.596 | -13.727 | -26.262 | -16.342 | -18.860 | -15.693 | -23.104 | -20.328 |
|  | 0.713 | -1.550 | -5.878 | -3.898 | -0.639 | 0.407 | 0.330 | -3.069 | -6.718 | -2.818 | -3.068 | -3.992 | -3.628 | 3.665 |
| **Sample estimates: mean of x (EN), mean of y (LN)** | 2.045 | 22.579 | 52.532 | 33.297 | 10.405 | 0.544 | 51.611 | 6.752 | 43.898 | 83.336 | 127.399 | 5.774 | 71.648 | 124.302 |
|  | 3.442 | 31.991 | 71.369 | 44.422 | 14.738 | 0.610 | 61.744 | 15.150 | 60.387 | 92.916 | 138.363 | 15.617 | 85.014 | 132.633 |
| **Percentage change** | 63% | -35% | 36% | 33% | 42% | 12% | 20% | 124% | 38% | 11% | 9% | 170% | 19% | 7% |
| **Effect size** | -0.249 | -0.450 | -0.549 | -0.579 | -0.440 | -0.052 | -0.365 | -0.588 | -0.632 | -0.532 | -0.529 | -0.626 | -0.516 | -0.260 |
| **Magnitude** | Small | Small | **Medium** | **Medium** | Small | Negligible | Small | **Medium** | **Medium** | **Medium** | **Medium** | **Medium** | **Medium** | Small |

| **Table S2:** Impacts of specific El Niño events. Noteworthy results are highlighted by bold text. | | | | | | | | | | | | | | |
| --- | --- | --- | --- | --- | --- | --- | --- | --- | --- | --- | --- | --- | --- | --- |
|  | **Subtropical AEZs** | | | | | | | **Tropical AEZs** | | | | | | |
|  | **Cool-arid** | **Cool-semiarid** | **Warm-humid** | **Warm-subhumid** | **Warm-semiarid** | **Warm-arid** | **Cool-subhumid** | **Warm-arid** | **Warm-semiarid** | **Warm-subhumid** | **Warm-humid** | **Cool-arid** | **Cool-semiarid** | **Cool-subhumid** |
| **El Niño 2015/2016** | | | | | | | | | | | | | | |
| **T-stat** | 3.572 | 1.960 | 1.655 | 2.884 | 3.101 | 2.609 | 0.695 | 0.431 | 0.595 | 1.12 | 0.703 | 2.079 | 0.482 | -0.035 |
| **Degrees of freedom** | 55.436 | 20.715 | 18.555 | 24.033 | 26.683 | 76.818 | 14.722 | 14.575 | 16.62 | 17.31 | 15.171 | 23.381 | 15.555 | 20.119 |
| **P-value** | **0.0007**** | 0.064 | 0.114 | **0.008*** | **0.005*** | **0.011*** | 0.497 | 0.672 | 0.559 | 0.278 | 0.492 | **0.048*** | 0.636 | 0.971 |
| **95^th^ % confidence interval** | 1.755 | -0.554 | -3.771 | 3.392 | 2.531 | 0.203 | -12.538 | -5.901 | -11.091 | -5.690 | -12.468 | 0.030 | -14.472 | -22.716 |
|  | 6.239 | 18.369 | 32.019 | 20.462 | 12.450 | 1.519 | 24.645 | 8.885 | 19.803 | 18.605 | 24.767 | 10.366 | 22.970 | 21.952 |
| **Sample estimates: mean of x (N), mean of y (EN)** | 5.185 | 29.225 | 62.347 | 41.633 | 16.880 | 1.132 | 51.713 | 9.445 | 45.008 | 84.160 | 132.568 | 10.407 | 68.066 | 113.593 |
|  | 1.188 | 20.317 | 48.222 | 29.707 | 9.390 | 0.271 | 45.660 | 7.952 | 40.652 | 77.703 | 126.419 | 5.209 | 63.817 | 113.976 |
| **Percentage change** | -77% | -30% | -23% | -29% | -44% | -76% | -12% | -16% | -10% | -8% | -5% | -50% | -6% | 0% |
| **Effect size** | 0.564 | 0.453 | 0.414 | 0.613 | 0.626 | 0.339 | 0.219 | 0.138 | 0.163 | 0.296 | 0.213 | 0.448 | 0.142 | -0.009 |
| **Magnitude** | **Medium** | Small | Small | **Medium** | **Medium** | Small | Small | Negligible | Negligible | Small | Small | Small | Negligible | Negligible |
| **El Niño 1997/1998** | | | | | | | | | | | | | | |
| **T-stat** | 1.020 | 0.071 | 0.001 | -0.311 | -0.056 | 1.151 | -0.148 | 2.991 | 0.537 | 0.0134 | 0.543 | 3.508 | 0.082 | -0.377 |
| **Degrees of freedom** | 7.590 | 5.694 | 5.553 | 5.567 | 5.537 | 15.132 | 5.524 | 15.243 | 6.418 | 5.883 | 6.409 | 19.213 | 6.005 | 6.099 |
| **P-value** | 0.338 | 0.945 | 0.999 | 0.766 | 0.957 | 0.268 | 0.887 | **0.009*** | 0.609 | 0.990 | 0.605 | **0.002*** | 0.937 | 0.719 |
| **95^th^ % confidence interval** | -2.666 | -23.053 | -45.658 | -29.430 | -16.934 | -0.475 | -38.690 | 1.641 | -17.360 | -22.924 | -18.487 | 2.838 | -28.247 | -47.302 |
|  | 6.832 | 24.422 | 45.728 | 22.888 | 16.187 | 1.592 | 34.348 | 9.739 | 27.331 | 23.175 | 29.249 | 11.220 | 30.196 | 34.637 |
| **Sample estimates: mean of x (N), mean of y (EN)** | 5.185 | 29.225 | 62.346 | 41.633 | 16.880 | 1.133 | 51.713 | 9.445 | 45.008 | 84.160 | 132.568 | 10.407 | 68.066 | 113.593 |
|  | 3.102 | 28.540 | 62.312 | 44.905 | 17.253 | 0.574 | 53.885 | 3.754 | 40.022 | 84.035 | 127.188 | 3.378 | 67.092 | 119.925 |
| **Percentage change** | -40% | -2% | 0% | +8% | +2% | -49% | +4% | -60% | -11% | 0% | -4% | -68% | -1% | +6% |
| **Effect size** | 0.281 | 0.0331 | 0.001 | -0.157 | -0.029 | 0.210 | -0.077 | 0.546 | 0.186 | 0.006 | 0.188 | 0.596 | 0.032 | -0.146 |
| **Magnitude** | Small | Negligible | Negligible | Negligible | Negligible | Small | Negligible | **Medium** | Negligible | Negligible | Negligible | **Medium** | Negligible | Negligible |
| **El Niño 1991/1992** | | | | | | | | | | | | | | |
| **T-stat** | 4.508 | 7.016 | 2.498 | 2.747 | 3.972 | 1.286 | 0.145 | 6.759 | 1.487 | 0.894 | 0.132 | 5.582 | 0.319 | -0.170 |
| **Degrees of freedom** | 50.055 | 38.44 | 6.790 | 6.686 | 12.501 | 30.78 | 7.102 | 74.429 | 7.016 | 7.513 | 0.132 | 61.554 | 6.145 | 6.485 |
| **P-value** | **<0.001**** | **<0.001**** | **0.042*** | **0.030*** | **0.002*** | 0.208 | 0.1459 | **<0.001**** | 0.180 | 0.399 | 0.899 | **<0.001**** | 0.760 | 0.870 |
| **95^th^ % confidence interval** | 2.554 | 14.450 | 1.295 | 2.339 | 4.356 | -0.304 | -5.810 | 6.151 | -7.049 | -8.754 | -24.845 | 5.665 | -23.869 | -38.080 |
|  | 6.659 | 26.165 | 53.506 | 33.415 | 14.834 | 1.340 | 31.986 | 11.292 | 30.975 | 19.643 | 27.705 | 11.985 | 31.066 | 33.049 |
| **Sample estimates: mean of x (N), mean of y (EN)** | 5.185 | 29.225 | 62.347 | 41.633 | 16.880 | 1.132 | 51.713 | 9.445 | 45.008 | 84.160 | 132.568 | 10.407 | 68.066 | 113.593 |
|  | 0.579 | 8.917 | 34.946 | 23.757 | 7.285 | 0.614 | 38.625 | 0.723 | 33.045 | 78.716 | 131.138 | 1.582 | 64.468 | 116.109 |
| **Percentage change** | -89% | -69% | -44% | -43% | -57% | -46% | -25.3% | -92% | -27% | -6% | -1% | -85% | -5% | +2% |
| **Effect size** | 0.629 | 1.026 | 0.790 | 0.889 | 0.784 | 0.196 | 0.219 | 0.841 | 0.450 | 0.249 | 0.050 | 1.025 | 0.120 | -0.058 |
| **Magnitude** | **Medium** | **Large** | **Medium** | **Large** | **Medium** | Negligible | Small | **Large** | Small | Small | Negligible | **Large** | Negligible | Negligible |

| **Table S3:** Impacts of specific La Niña events. Noteworthy results are highlighted by bold text. | | | | | | | | | | | | | | |
| --- | --- | --- | --- | --- | --- | --- | --- | --- | --- | --- | --- | --- | --- | --- |
|  | **Subtropical AEZs** | | | | | | | **Tropical AEZs** | | | | | | |
|  | **Cool-arid** | **Cool-semiarid** | **Warm-humid** | **Warm-subhumid** | **Warm-semiarid** | **Warm-arid** | **Cool-subhumid** | **Warm-arid** | **Warm-semiarid** | **Warm-subhumid** | **Warm-humid** | **Cool-arid** | **Cool-semiarid** | **Cool-subhumid** |
| **La Niña 2010/2012** | | | | | | | | | | | | | | |
| **T-stat** | 1.338 | 0.136 | 0.633 | 1.153 | 2.268 | 1.949 | -0.189 | -0.336 | -1.610 | -1.011 | -1.668 | -1.877 | -3.114 | -2.145 |
| **Degrees of freedom** | 19.102 | 16.044 | 16.588 | 17.265 | 25.283 | 73.34 | 21.087 | 14.092 | 14.104 | 19.209 | 28.367 | 12.005 | 17.438 | 18.429 |
| **P-value** | 0.197 | 0.893 | 0.535 | 0.264 | **0.032*** | 0.055 | 0.851 | 0.742 | 0.130 | 0.325 | 0.106 | 0.085 | **0.006*** | **0.046*** |
| **95^th^ % confidence interval** | -1.257 | -10.291 | -12.837 | -4.634 | 0.499 | -0.016 | -12.895 | -7.822 | -29.881 | -15.201 | -19.251 | -19.542 | -37.067 | -42.242 |
|  | 5.718 | 11.704 | 23.826 | 15.820 | 10.276 | 1.437 | 10.743 | 5.703 | 4.242 | 5.296 | 1.963 | 1.454 | -7.159 | -0.471 |
| **Sample estimates: mean of x (N), mean of y (LN)** | 5.185 | 29.225 | 62.347 | 41.634 | 16.880 | 1.132 | 51.713 | 9.445 | 45.008 | 84.160 | 132.568 | 10.407 | 68.066 | 113.593 |
|  | 2.954 | 28.518 | 56.852 | 36.041 | 11.492 | 0.422 | 52.789 | 10.504 | 57.827 | 89.113 | 141.213 | 19.451 | 90.179 | 134.950 |
| **Percentage change** | -43% | -2% | -9% | -13% | -32% | -63% | +2% | +11% | +28% | +6% | +7% | +87% | +32% | +19% |
| **Effect size** | 0.307 | 0.036 | 0.161 | 0.283 | 0.450 | 0.277 | -0.041 | -0.010 | -0.479 | -0.231 | -0.315 | -0.717 | -0.760 | -0.507 |
| **Magnitude** | Small | Negligible | Negligible | Small | Small | Small | Negligible | Negligible | Small | Small | Small | **Medium** | **Medium** | **Medium** |
| **La Niña 2007/2009** | | | | | | | | | | | | | | |
| **T-stat** | 1.170 | 0.103 | 0.2385 | 0.591 | 2.455 | 1.739 | -1.798 | -1.344 | -2.020 | -1.497 | -0.522 | -0.927 | -2.017 | -1.842 |
| **Degrees of freedom** | 15.709 | 14.478 | 17.436 | 15.888 | 22.173 | 68.706 | 18.556 | 11.946 | 13.071 | 15.041 | 17.921 | 10.763 | 13.443 | 16.12 |
| **P-value** | 0.259 | 0.918 | 0.814 | 0.562 | **0.022*** | **0.087*** | 0.088 | 0.204 | 0.064 | 0.155 | 0.608 | 0.374 | 0.064 | 0.084 |
| **95^th^ % confidence interval** | -1.868 | -11.812 | -15.522 | -8.001 | 0.968 | -0.095 | -24.041 | -15.184 | -37.412 | -21.967 | -17.476 | -23.529 | -39.582 | -44.068 |
|  | 6.453 | 13.019 | 19.489 | 14.182 | 11.478 | 1.392 | 1.845 | 3.603 | 1.247 | 3.842 | 10.519 | 9.611 | 1.296 | 3.078 |
| **Sample estimates: mean of x (N), mean of y (LN)** | 5.185 | 29.225 | 62.347 | 41.634 | 16.880 | 1.132 | 51.713 | 9.445 | 45.008 | 84.160 | 132.568 | 10.407 | 68.065 | 113.593 |
|  | 2.892 | 28.622 | 60.364 | 38.543 | 10.657 | 0.484 | 62.812 | 15.235 | 63.090 | 93.223 | 136.047 | 17.366 | 87.209 | 134.089 |
| **Percentage change** | -44% | -2% | -3% | -7% | -37% | -57% | +21% | +61% | +40% | +11% | +3% | +67% | +28% | +18% |
| **Effect size** | 0.311 | 0.030 | 0.058 | 0.156 | 0.517 | 0.252 | -0.420 | -0.519 | -0.665 | -0.415 | -0.125 | -0.486 | -0.637 | -0.482 |
| **Magnitude** | Small | Negligible | Negligible | Negligible | **Medium** | Small | Small | **Medium** | **Medium** | Small | Negligible | Small | **Medium** | Small |
| **La Niña 1998/2001** | | | | | | | | | | | | | | |
| **T-stat** | 1.797 | 0.700 | -1.163 | -0.987 | 0.129 | 1.901 | -1.139 | -0.845 | -1.734 | -1.541 | -0.0259 | -0.298 | -1.605 | -1.794 |
| **Degrees of freedom** | 37.098 | 24.193 | 22.399 | 24.378 | 25.026 | 84.344 | 21.255 | 17.174 | 19.641 | 20.84 | 30.341 | 17.501 | 20.751 | 5.163 |
| **P-value** | 0.080 | 0.490 | 0.257 | 0.333 | 0.897 | 0.060 | 0.267 | 0.410 | 0.098 | 0.138 | 0.979 | 0.769 | 0.123 | 0.131 |
| **95^th^ % confidence interval** | -0.326 | -7.001 | -31.063 | -15.520 | -5.912 | -0.030 | -25.687 | -14.503 | -34.025 | -24.165 | -12.559 | -12.551 | -33.395 | -64.825 |
|  | 5.441 | 14.192 | 8.731 | 5.472 | 6.707 | 1.348 | 7.498 | 6.204 | 3.149 | 3.599 | 12.244 | 9.435 | 4.314 | 11.246 |
| **Sample estimates: mean of x (N), mean of y (LN)** | 5.185 | 29.225 | 62.347 | 41.634 | 16.880 | 1.132 | 51.713 | 9.445 | 45.008 | 84.160 | 132.568 | 10.407 | 68.066 | 113.593 |
|  | 2.628 | 25.629 | 73.513 | 46.658 | 16.483 | 0.474 | 60.808 | 13.594 | 60.446 | 94.443 | 132.726 | 11.965 | 82.606 | 140.383 |
| **Percentage change** | -49% | -12% | +18% | +12% | -2% | -58% | +18% | +44% | +34% | +12% | 0% | +15% | +21% | +24% |
| **Effect size** | 0.359 | 0.179 | -0.317 | -0.250 | 0.032 | 0.265 | -0.327 | -0.329 | -0.545 | -0.451 | -0.006 | -0.112 | -0.473 | -0.620 |
| **Magnitude** | Small | Negligible | Small | Small | Negligible | Small | Small | Small | **Medium** | Small | Negligible | Negligible | Small | **Medium** |
